# Papillomavirus-like particles as vectors for *ex vivo* gene therapy of the skin

**DOI:** 10.1016/j.omtn.2025.102501

**Published:** 2025-03-05

**Authors:** Francesco Diversi, Juliette Dabin, Elisa Mazza, Mirko Rinaldin, Fernanda de Castro Reis, Jamie A. Hackett, Paul A. Heppenstall

**Affiliations:** 1 Neuroscience Area, International School for Advanced Studies (SISSA/ISAS), Via Bonomea 265, 34136 Trieste, Italy; 2 Epigenetics & Neurobiology Unit European Molecular Biology Laboratory (EMBL) Rome, Italy

**Keywords:** MT: Delivery Strategies, Papillomavirus, viral vector, virus like particles, Olmsted syndrome, Trpv3, skin gene therapy

## Abstract

*Ex vivo* gene delivery to the skin utilizing retroviral vectors has been demonstrated to be a viable clinical option for replacement of defective genes. However, because these vectors integrate their cargo into the genome, safety issues arise when utilizing them to deliver gene-editing nucleases. Here, we explored the use of Papillomavirus, a non-integrating viral vector, for *ex vivo* skin gene editing, exploiting its natural tropism for basal keratinocytes. We demonstrated that Papillomavirus-like particles (PVLPs) can deliver a variety of DNA constructs encoding fluorophores, Cre recombinase, calcium indicators, Cas9, and short hairpin RNA (shRNA) to keratinocytes, offering advantages over other viral vectors such as adeno-associated virus (AAV) and Lentivirus. We further showed that PVLPs can be used for gene therapy for Olmsted syndrome, a genetic skin disease caused by a gain-of-function mutation in the *Trpv3* gene. Specifically, PVLP-delivered SaCas9 and shRNA effectively disrupted the *Trpv3* gene or reduced its expression, leading to decreased TRPV3 activity and mitigating the hyperactivity associated with Olmsted syndrome. Skin equivalents generated from PVLP-treated keratinocytes exhibited complete transduction, and PVLP-shRNA treatment significantly reduced hyperkeratosis in skin equivalents from mice bearing the Olmsted syndrome mutation. These findings highlight PVLP as a promising tool for *ex vivo* skin gene therapy.

## Graphical abstract

**Figure:**
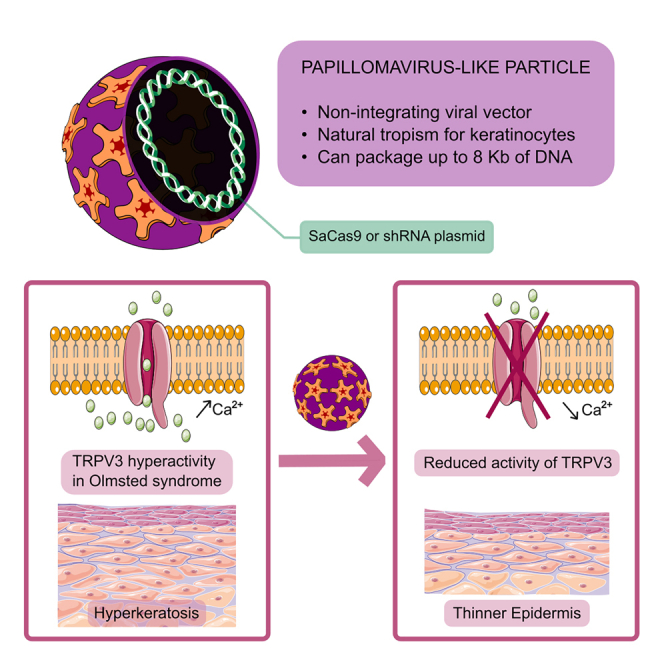

## Teaser

Diversi and colleagues demonstrate that Papillomavirus-like particle (PVLP), a non-integrating viral vector, enables *ex vivo* gene therapy in keratinocytes. PVLPs deliver gene-editing tools to treat Olmsted syndrome, reducing TRPV3 hyperactivity and hyperkeratosis. This study highlights PVLP as a promising viral vector for skin gene therapy.

## Introduction

Olmsted syndrome is a rare dominant genetic skin disease caused by a gain-of-function point mutation in the transient receptor potential vanilloid-3 (*Trpv3*) gene (Ensembl: ENSMUSG00000043029).^1^ TRPV3 is a non-selective cation channel sensitive to non-noxious warm temperatures (31°C–39°C) and mainly expressed in keratinocytes and sensory neurons.^2^ The mutation renders the channel hyperactive,^3^ thus increasing intracellular calcium levels; this leads to hyperproliferation and defects in the maturation of keratinocytes, invasion of immune cells, and chronic itch and pain.^1^^,^^4^^,^^5^^,^^6^ Olmsted syndrome, given its well-defined etiology and the general innocuity of *Trpv3* KO in mice,^7^^,^^8^ stands out as a promising target for gene therapy. However, targeting the epidermis for gene therapy poses considerable challenges: skin is the largest organ in the human body, and it displays a strong primary defense against entry of viral vectors, both because of its barrier nature and the presence of resident immune cells.

Here, we considered Papillomavirus as a new viral vector for treating genetic skin diseases such as Olmsted syndrome. Papillomavirus has a natural tropism for continuously dividing basal keratinocytes, as it readily infects actively dividing cells by entering the nucleus only during mitosis.^9^^,^^10^ Papillomavirus is a non-enveloped virus composed of major L1 and minor L2 capsid proteins and can package up to 8 kb of double-stranded DNA that typically remains episomal upon cellular delivery.^11^ These characteristics make Papillomavirus a promising candidate for delivering gene therapies based on genome editing. It can deliver large constructs, such as base editors or large Cas9 proteins, which exceed the 4.7-kb packing limit of adeno-associated viruses (AAVs). It also has an advantage over lentiviruses, as it does not integrate its cargo into the host genome, thus avoiding unintended off-target effects, as well as the risk of disrupting host genes. Moreover, recombinant Papillomavirus-like particles (PVLPs) can be produced at high yields by simply transfecting HEK293TT cells with a plasmid encoding the capsid proteins together with the desired cargo plasmid^11^ and in a cell-free reaction by incubating purified capsid proteins with double-stranded DNA fragments smaller than 8 kb.^12^^,^^13^

In this study, we employed PVLP to deliver either the Cas9 nuclease from *Staphylococcus aureus* (SaCas9) with a guide RNA (gRNA) to disrupt *Trpv3* or a short hairpin RNA (shRNA) to interfere with *Trpv3* expression. We demonstrated efficient transduction of a mouse keratinocyte cell line (KERA-308) and mouse primary keratinocytes by PVLP that compared favorably to other commonly used viral vectors, AAVs and lentiviruses. Moreover, in skin equivalents derived from PVLP-treated primary keratinocytes,^14^ we observed almost complete transduction of keratinocytes by PVLPs, supporting their applicability for generation of autologous transgenic skin transplants. Finally, targeting *Trpv3* via PVLP delivery of SaCas9 or shRNA was remarkably efficient in primary keratinocytes and resulted in rescue of Olmsted syndrome phenotypes in *ex vivo* mouse skin equivalents.

## Results

### Papillomavirus as a viral vector

We first compared transduction efficiency of cell-assembled versus cell-free-assembled PVLP for both human Papillomavirus 16 (HPV16)- and murine Papillomavirus 1 (MuPV1)-derived capsids (referred to as human papillomavirus-like particle [HPVLP] and murine papillomavirus-like particle [MuPVLP]).^15^^,^^16^^,^^17^^,^^18^ Using a fluorescent reporter as cargo, these four combinations were tested in three different cell types: a human cell line (HEK293 cells), a murine keratinocyte cell line (KERA-308), and primary keratinocytes from mice.

**Figure 1:**
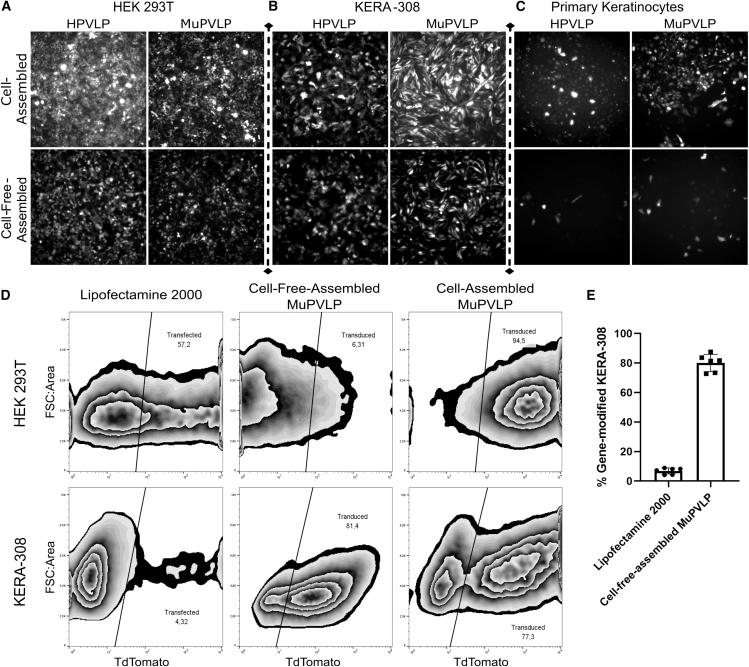
HPVLP and MuPVLP were used to encapsidate a TdTomato plasmid via cell assembly or by cell-free assembly The fluorescent reporter was employed to evaluate the transduction efficiency of HPVLP and MuPVLP in HEK-293T (A), KERA-308 (B), or in primary murine keratinocytes (C) at 3 days post infection. (D) Flow cytometry comparison of the transfection efficiency of Lipofectamine 2000, cell-free-assembled MuPVLP (2 μg of encapsidated DNA), and cell-assembled MuPVLP 5000 MOI in HEK-293T and KERA-308 5 dpi. (E) Quantification of the efficiency of Lipofectamine 2000 and of cell-free-assembled MuPVLP in KERA-308 cells (*N* = 6).

As expected, in human HEK293 cells, HPVLPs showed higher transduction efficiency than MuPVLPs (Figure 1A), while, in mouse KERA-308 cells, MuPVLP performed better than HPVLP (Figure 1B). In both cell lines, those transduced by cell-assembled PVLPs displayed consistently higher levels of fluorescence.

In primary murine keratinocytes, cell-assembled MuPVLP performed better than cell-assembled HPVLP (Figure 1C). However, cell-free-assembled PVLPs of both serotypes failed to efficiently transduce these primary cells, likely due to the absence of some undefined cell-specific nuclear factors in the *in vitro* reaction.^12^^,^^13^ Thus, because MuPVLP proved to be more efficient on murine cells and our target cells were mouse keratinocytes, we selected this capsid for further experiments.

While cell lines can usually be transfected easily using non-viral methods such as lipofection, we observed that KERA-308 cells were difficult to transfect. To determine whether PVLPs offered an advantage over this transfection method, we compared cell-free-assembled MuPVLPs to the commonly used Lipofectamine 2000 reagent (Figure 1D). Flow cytometry quantification showed that cell-free-assembled MuPVLPs had a significantly higher transfection efficiency in KERA-308 cells (but not HEK293 cells) compared to Lipofectamine 2000. Specifically, Lipofectamine 2000 transfected 6.8% ± 2.0% SD of KERA-308 cells, whereas cell-free-assembled MuPVLPs achieved a transduction rate of 80.0% ± 5.8% SD (Figure 1E).

We concluded that, while, upon visual inspection, KERA-308 treated with cell-free-assembled PVLP appeared to be less transduced than KERA-308 treated with cell-assembled PVLP (Figure 1B), this could be due to a higher number of particles infecting each cell. Nevertheless, most HEK and KERA-308 cells were still transduced by cell-free-assembled PVLPs, albeit with lower intensity, as shown by flow cytometry (Figures 1D and 1E).

Thus, for *in vitro* experiments on KERA-308 cells, we opted to use cell-free-assembled PVLPs, allowing us to rapidly test many cargo plasmids without the necessity of producing new PVLP batches in HEK293TT. Once optimal cargo configurations were identified, we cloned the SV40 promoter into the plasmid and then used cell-assembled MuPVLPs for experiments on primary keratinocytes.

### Comparison of different viral vectors in transducing primary keratinocytes

We next compared the transduction efficiency of PVLP in primary keratinocytes with that of other commonly used viral vectors, AAV and Lentivirus. To determine which AAV serotype to use, we compared the transduction efficiency of AAV1, 2, 3, 5, 8, and 9 in primary keratinocytes 5 days post infection at 10^5^ multiplicity of infection (MOI) (Figure S1). AAV1/2 exhibited the highest transduction rates, aligning with the results of Ellis et al.^19^ in their comparative study. Based on these findings, we decided to proceed with the AAV1/2 serotype for further experiments. Regarding the Lentivirus choice, we opted to use a standard integrating Lentivirus in our comparison experiment, rather than a non-integrating variant, because non-integrating lentiviruses (NILVs) generally exhibit lower transduction efficiency.^20^ Our aim was to evaluate and compare the transduction potential against the most effective available method, making the integrating Lentivirus a more appropriate choice. This approach allowed us to set a higher benchmark for efficiency, ensuring a more meaningful assessment of the transduction capabilities of the alternative methods.

For comparing the different viral vectors in transducing primary keratinocytes, we employed primary cultures of keratinocytes derived from a Lox-STOP-Lox-TdTomato (LSL) transgenic mouse that were transduced with MuPVLP, Lentivirus, or AAV1/2 carrying a Cre recombinase cargo at different MOI (Figure S2).

**Figure 2:**
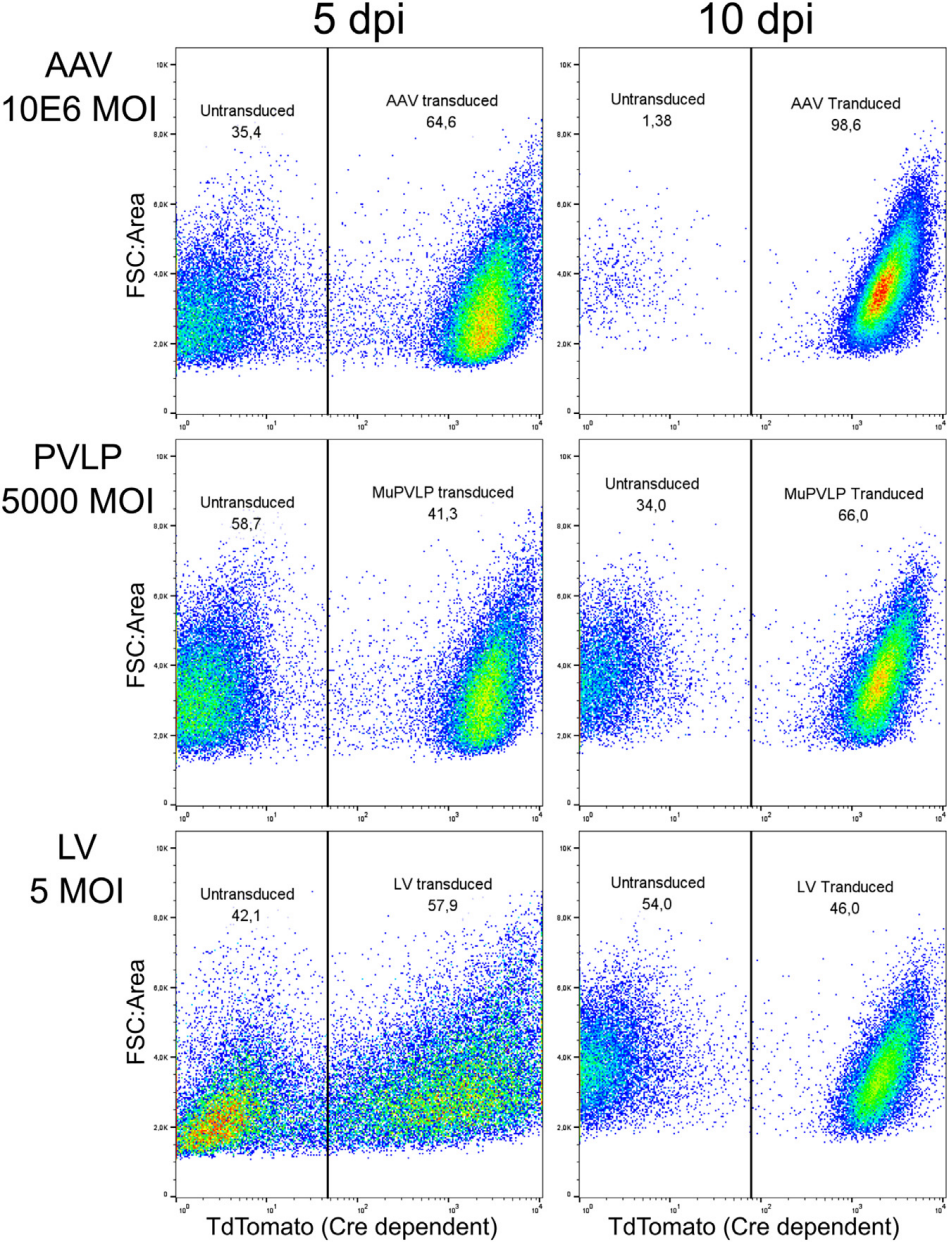
Comparison via flow cytometry of the transduction efficiency of MuPVLP, Lentivirus and AAV1/2 at their highest effective Multiplicity Of Infection (MOI) LSL-TdTomato mouse primary keratinocytes were transduced with different MOI of the three viral vectors delivering a Cre construct. At 5 and 10 dpi, the primary keratinocytes were analyzed with flow cytometry for TdTomato expression.

The efficiency of transduction was quantified via expression of TdTomato using a quantitative flow cytometry assay 5 days post infection and 10 days post infection (Figure 2), while cytotoxicity was evaluated through eFluor450 incorporation (Figure S3). At 5 days post infection, MuPVLP displayed a lower transduction efficiency with respect to Lentivirus or AAV (Figure 2A) but was less cytotoxic than Lentivirus (Figure S3). However, MuPVLP also demonstrated cytotoxicity at higher MOI (Figure S3). AAV showed the highest transduction efficiency, but only at a high MOI (10^6^), exceeding the range typically used for cell transduction (10^4^–10^5^).

At 10 days post infection, MuPVLP performed better than Lentivirus, with 66% of the primary keratinocytes transduced by MuPVLP against the 46% transduced by Lentivirus (Figure 2B). Consequently, MuPVLP was used at an MOI of 5,000 for all subsequent experiments. We hypothesize that MuPVLP’s superior performance over a longer period may stem from its ability to transduce only dividing cells as reported previously,^21^^,^^22^ which eventually dominated the culture. Overall, these results indicate that PVLP may be an optimal vector for gene-editing approaches in dividing cells, offering higher transduction efficiency, reduced cytotoxicity compared to Lentivirus, and a larger cargo capacity than AAV.

### SaCas9 delivered via MuPVLP disrupts the Trpv3 locus in KERA-308 and primary keratinocytes

To investigate the potential of PVLP as a vector for skin gene therapy, we used it to deliver SaCas9 and a gRNA to disrupt the *Trpv3* gene. We quantified the disruption of the *Trpv3* locus both using sequencing of genomic DNA and with a functional assay based upon calcium imaging of cells stimulated with a Trpv3 agonist.

**Figure 3:**
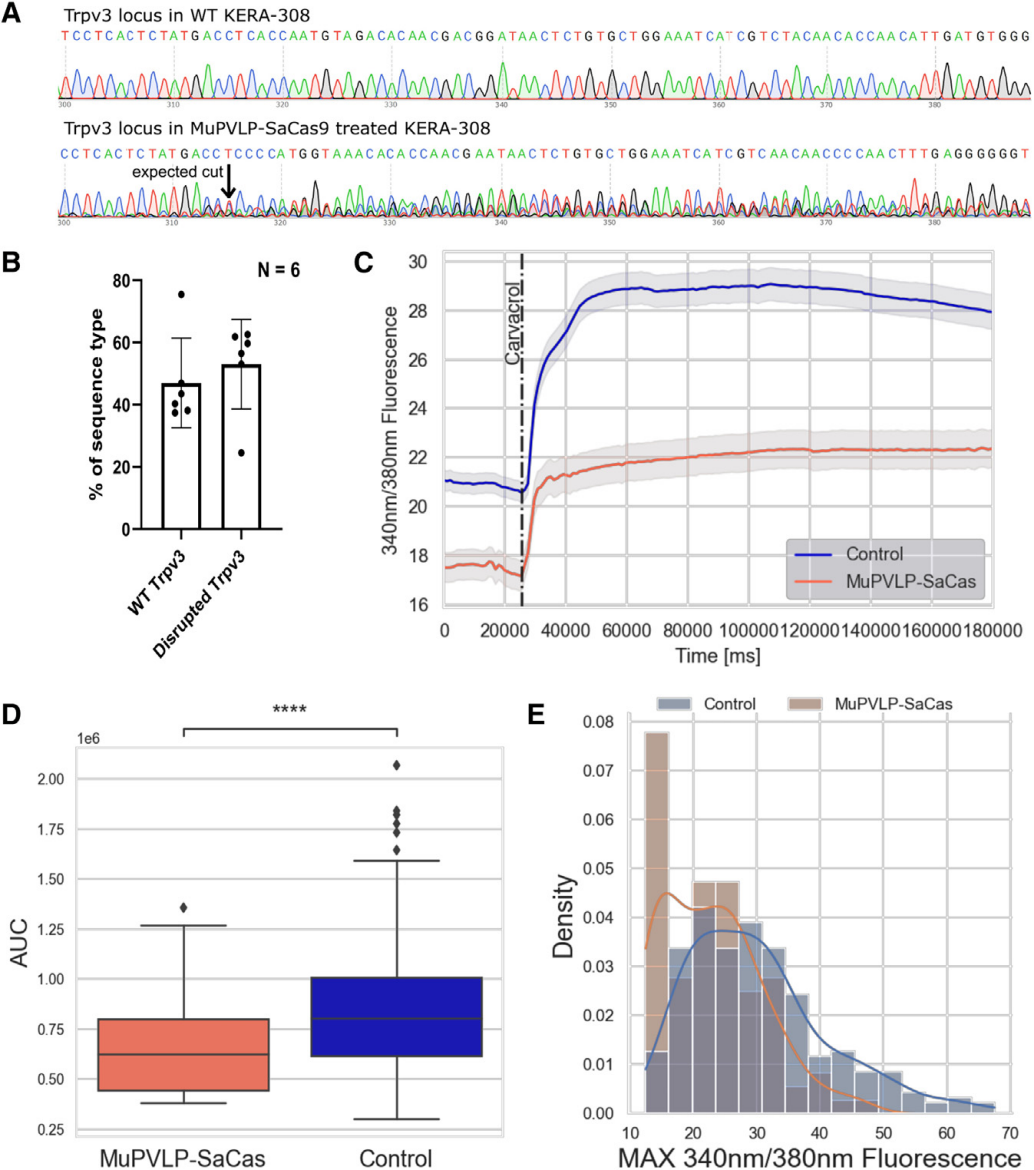
Validation of Trpv3 gene disruption and TRPV3 function after MuPVLP-SaCas9 treatment in KERA-308 cells (A) Chromatogram of Trpv3 around the expected cut site before and after MuPLVP-SaCas9 treatment. (B) Percentage of indels detected via TIDE analysis of the Trpv3 gene in MuPVLP-SaCas9-treated KERA-308 cells (*N* = 6). (C) FURA2-AM 340/380 nm fluorescence average response ±SEM of control and MuPVLP-SaCas9-treated KERA-308 to carvacrol. Control KERA-308 *N* = 258; MuPVLP-SaCas treated KERA-308 *N* = 98. (D) Mann-Whitney-Wilcoxon test two-sided with Bonferroni correction statistical analysis of the area under the curve of the two groups of cells in the 30 s after the application of carvacrol. *p* = 8.019e−08. (E) Density plot of the maximal 340/380-nm fluorescence in response to carvacrol of the two groups.

We selected the gRNA that provoked the highest indel formation out of three candidate gRNAs (Figure S4). This gRNA targets exon 9, while the Olmsted mutation is in exon 13; we opted for a gRNA in the core of the protein so that it could disrupt the channel functionality. We cloned the selected gRNA into a plasmid with SaCas9-T2A-mCherry to visualize the cells that incorporated the construct. Then we produced cell-free-assembled MuPVLPs and transduced KERA-308. After 10 days, we extracted the genomic DNA from all KERA-308 to also consider untransduced cells. We sequenced the targeted Trpv3 locus and analyzed the indel formation via Tracking of Indels by DEcomposition (TIDE). On average, about half of the alleles were disrupted, usually because of the deletion of one or two nucleotides (Figures 3A and 3B).

We performed a western blot using a TRPV3 antibody. In cells treated with PVLP-SaCas9, we detected a band with lower molecular weight than the expected 91-kDa wild-type (WT) TRPV3, which may correspond to a truncated TRPV3 variant provoked by the gene disruption. We also found a reduction in the intensity of the expected WT 91-kDa band in the PVLP-SaCas9-treated KERA-308 (Figure S5).

To understand the efficacy of the SaCas9, we co-transfected KERA-308 cell with a plasmid containing SaCas9, gRNA, and a hygromycin-resistance cassette enclosed in transposon-specific Inverted Terminal Repeats (ITRs), and a PiggyBac plasmid to integrate the first plasmid in the KERA-308 genome. After 2 weeks of hygromycin selection, we harvested the cells and analyzed the disruption via TIDE. We found that the overall efficacy of SaCas9 in inducing indels was 60.5% (Figure S6), while the overall efficacy of SaCas9 delivered via PVLP was 53% (Figure 3B). This would indicate that the major cause of inefficiency was the Cas9 itself and not the delivery system, which we know from the comparative study with TdTomato to transduce between 70% and 80% of KERA-308 (Figure 1). We thus chose not to normalize the TIDE results for transduction efficiency, since the following calcium imaging experiments with FURA2-AM were performed without selecting the transduced cells, assuming as average transduction efficiency ∼80% for KERA-308 treated with cell-free-assembled MuPVLP (Figure 1) and ∼60% for primary keratinocytes treated with cell-assembled MuPVLP (Figure 2).

Next, we evaluated the functionality of the TRPV3 cation channel via calcium imaging. Since *Trpv3* encodes for a Ca^2+^-permeable non-selective cation channel, we tested its functionality by stimulating its opening with 500 μM TRPV3 agonist carvacrol.^23^ Although carvacrol is the most selective TRPV3 agonist, it can also activate the transient receptor potential ankyrin 1 (TRPA1) channel.^24^ For this reason, we included in the imaging medium 50 μM TRPA1 antagonist HC 030031 in all the experiments (Figure S7).^25^

In initial experiments, we used the genetically encoded calcium indicator GCaMP8s to monitor intracellular calcium flux (Figure S8). If the Trpv3 locus was disrupted by MuPVLP-SaCas9, we would expect a reduced response to the carvacrol stimulus, indicating that the channel is non-functional. We co-transduced KERA-308 with MuPVLP-SaCas9-mCherry and MuPVLP-GCaMP8s and, after 14 days, we monitored the response of the cells to the agonist carvacrol. Only cells that expressed both GCaMP8s and mCherry were analyzed in the treated group for calcium imaging (Figure S8A). KERA-308 treated with MuPVLP-SaCas9-mCherry responded significantly less to the agonist carvacrol (*p*= 3.7e−05), only triggering 60% of the normalized control response (Figures 8B–8D).

We further investigated TRPV3 functionality using the ratiometric dye FURA2-AM, which allowed us to make absolute measurements of calcium concentration rather than changes in calcium flux. We analyzed the global response of KERA-308 to carvacrol, without selecting mCherry-positive cells. On average, MuPVLP-SaCas9-treated KERA-308 had a lower baseline calcium level and responded significantly less (*p*= 8.0e−08); only upon stimulation with carvacrol did they reach the baseline on the untreated cells (Figures 3C and 3D). This reduced response presumably reflects the 6-fold increase in the carvacrol-unresponsive cell population (Figure 3E).

Next, we moved from the KERA-308 cell line to a more complex system evaluating the efficacy of SaCas9 delivered via MuPVLP in primary keratinocytes. We compared the effect of PVLP-SaCas9 on primary keratinocytes derived from either WT mice or from Dermatitis Speciosa-Non-Hair (DS-Nh) mice, a model of Olmsted syndrome. The DS-Nh mice bear the single point mutation G573S in the *Trpv3* gene,^26^ orthologous to the most typical SNP causing Olmsted syndrome in humans.^27^ The disease phenotype is passed down in a dominant fashion, so we used the mice heterozygous for Trpv3 to derive primary keratinocytes with the Olmsted genotype and as control primary keratinocytes derived from the homozygous WT DS-Nh mice.

**Figure 4:**
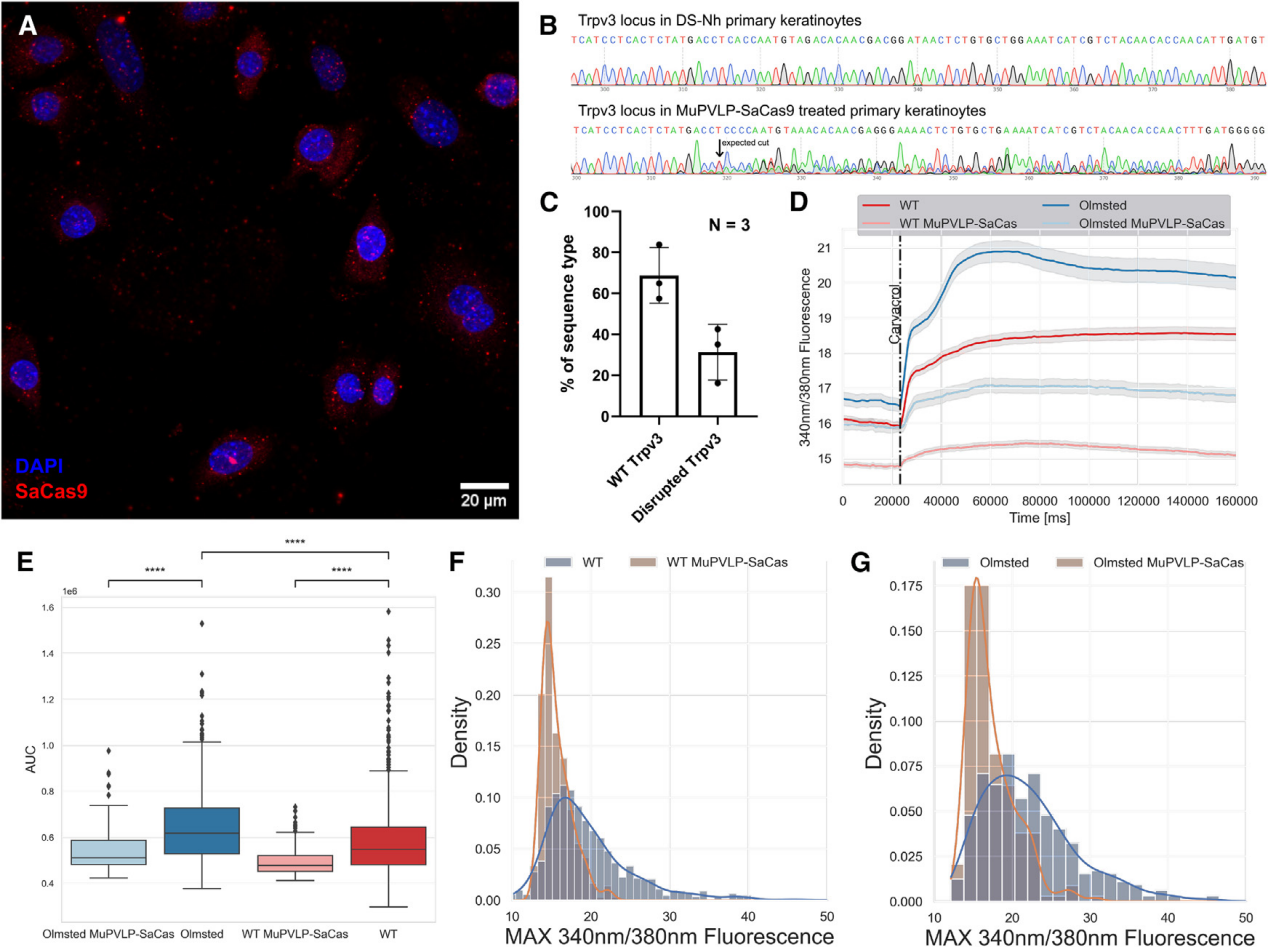
Validation of TRPV3 functional rescue after MuPVLP-SaCas9 treatment in a model of Olmsted syndrome (A) Immunocytochemistry against SaCas9 (red) and DAPI (blue). (B) Chromatogram of Trpv3 around the expected cut site before and after MuPLVP-SaCas9 treatment. (C) Percentage of indels detected via TIDE analysis of the Trpv3 gene in primary keratinocytes treated with MuPVLP-SaCas9 7 days post infection (*N* = 3). (D) Average FURA2-AM 340/380-nm fluorescence response ±SEM to carvacrol in control and MuPVLP-SaCas9-treated primary keratinocytes from wild-type (WT) or DS-Nh mice. Sample sizes: WT control (*N* = 1,012), WT MuPVLP-SaCas treated (*N* = 388), DS-Nh control (*N* = 396), DS-Nh MuPVLP-SaCas treated (*N* = 209). (E) Mann-Whitney-Wilcoxon test with Bonferroni correction comparing the area under the curve for the first 30 s after carvacrol application. WT MuPVLP-SaCas treated vs. WT control: *p* = 7.794e−34; DS-Nh MuPVLP-SaCas-treated vs. DS-Nh control: *p* = 4.671e−16; DS-Nh control vs. WT control: *p* = 1.228e−14. (F) Density plot of the maximal 340/380-nm fluorescence response to carvacrol comparing WT control and WT treated groups. (G) Density plot of the maximal 340/380-nm fluorescence response to carvacrol comparing DS-Nh control and DS-Nh treated groups.

MuPVLP was able to deliver SaCas9 to primary keratinocytes, as highlighted by its presence inside the nucleus via immunocytochemistry (Figure 4A), and it was able to disrupt the Trpv3 locus, albeit with less efficiency with respect to the cell line KERA-308 (Figures 4B and 4C). Functionally, we evaluated the collective calcium response to the agonist carvacrol both for WT and Olmsted primary keratinocytes. We observed that Olmsted primary keratinocytes responded more to the stimulus with respect to WT primary keratinocytes (Figure 4D), confirming the hyperexcitability state typical of the Olmsted syndrome.^3^ The primary keratinocytes treated with MuPVLP-SaCas9 responded significantly less to carvacrol compared to the corresponding untreated keratinocytes (Figures 4D and 4E). Importantly, Olmsted primary keratinocytes treated with MuPVLP-SaCas9 exhibited functional responses comparable with and marginally below WT levels, implying a rescue of the hyperexcitability by MuPVLP-SaCas9 delivery. This diminished response is reflected in the higher percentage of unresponsive keratinocytes (Figures 4F and 4G), suggesting an effective disruption of the *Trpv3* gene and the non-functionality of the channel.

Editing efficiency was approximately 50% in the KERA-308 cell line and 25% in primary keratinocytes. However, the observed reduction in channel functionality was unexpectedly higher than anticipated based on these editing levels. Although we did not investigate the presence of large deletions, this possibility could explain the disproportionately greater reduction in channel functionality. Taken together, these experiments on KERA-308 and primary keratinocytes demonstrated that MuPVLP can be used for delivery of SaCas9 plus gRNA and subsequent disruption of the selected Trpv3 gene. Indeed, the sequencing showed the presence of indels in Trpv3 locus, and the calcium imaging analysis suggested that the TRPV3 channel was hyperactive in heterozygote DS-Nh mice and non-functional after the disruption.

### shRNA delivered via MuPVLP silences Trpv3 in KERA-308 and primary keratinocytes

**Figure 5:**
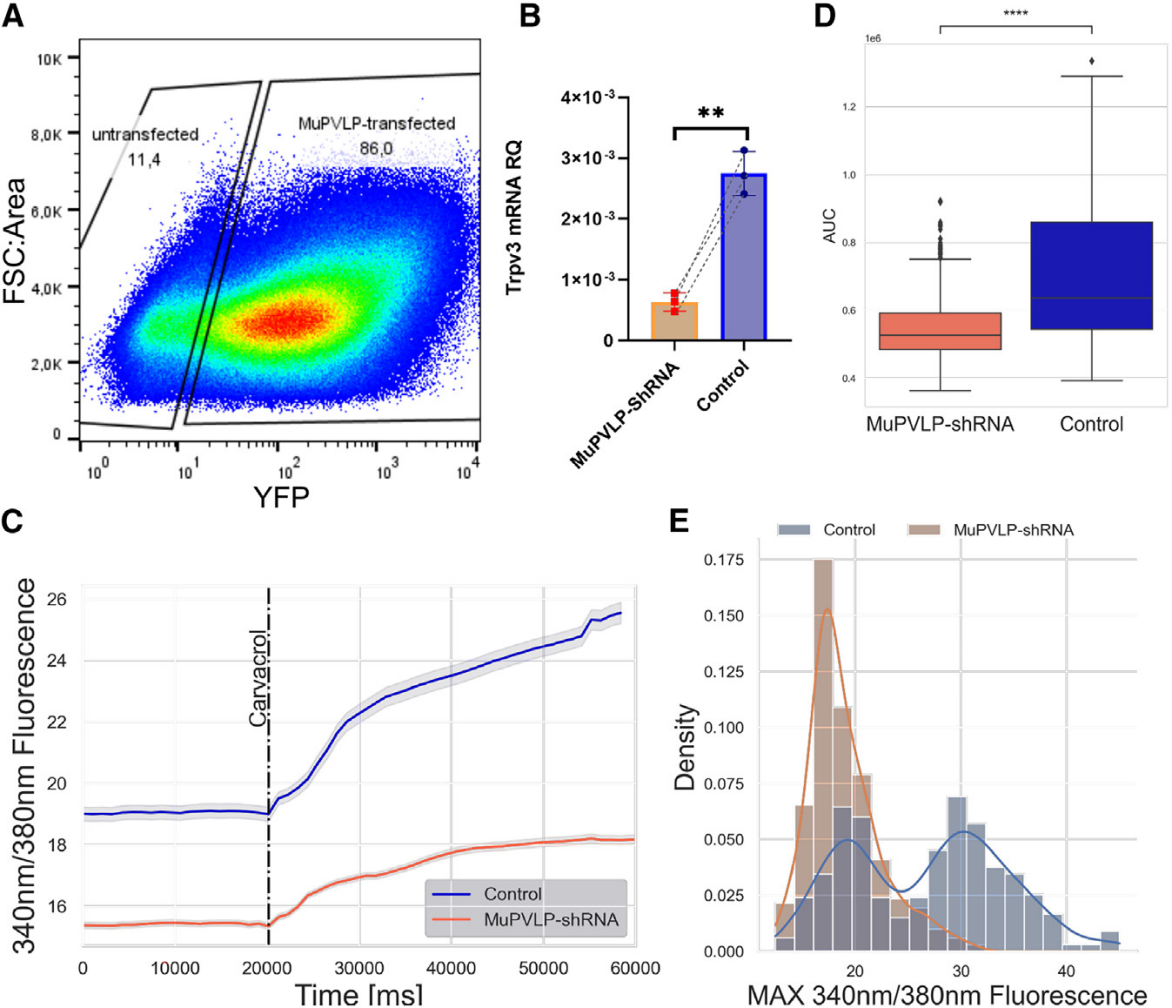
Validation of Trpv3 mRNA interference and reduced TRPV3 channel response after MuPVLP-shRNA-YFP treatment in KERA-308 cells (A) FACS strategy. (B) Relative quantification of Trpv3 mRNA compared to the housekeeping gene Gapdh via qPCR. Ratio paired t test of control (YFP− KERA-308, *N* = 3) vs. MuPVLP-shRNA-treated (YFP+ KERA-308, *N* = 3), one-tailed *p* = 0.0032. (C) Average FURA2-AM 340/380-nm fluorescence response ±SEM to carvacrol in control (*N* = 368) and MuPVLP-shRNA-treated (*N* = 567) KERA-308 cells. (D) Mann-Whitney-Wilcoxon test with Bonferroni correction comparing the area under the curve for the first 30 s after carvacrol application between the two groups. *p* = 1.973e−50. (E) Density plot of the maximal 340/380-nm fluorescence response to carvacrol in control and MuPVLP-shRNA-treated groups.

To investigate a more general applicability of PVLP as a vector for skin gene therapy, we used it to deliver a shRNA to silence Trpv3 mRNA via RNA interference. Four candidate shRNAs were cloned into a plasmid expressing YFP as a fluorescent reporter and delivered to KERA-308 via cell-free-assembled MuPVLP (Figure S9). Transduced KERA-308 were sorted via fluorescence-activated cell sorting (FACS) 5 days post treatment based on YFP expression (Figure 5A). The RNA was extracted from both the YFP+ and YFP− (control) populations and Trpv3 mRNA levels were quantified via relative qPCR with respect to GAPDH expression.^28^ Of the four shRNAs tested, one significantly reduced the expression of Trpv3 mRNA in KERA-308 cells to 23% ± 5% of that observed in the control cells (the other three were without effect) (Figures 5B and S9). We also tested the functionality of the TRPV3 channel in KERA-308 via FURA2-AM calcium imaging on the whole cell population, without selecting YFP-positive cells. MuPVLP-shRNA-treated KERA-308 responded significantly less (*p* = 1.9e−50) to the carvacrol stimulus (Figures 5C and 5D), reflecting a higher percentage of cells unresponsive to the TRPV3 agonist (Figure 5E). These data confirmed the knockdown effect of the shRNA delivered via MuPVLP.

**Figure 6:**
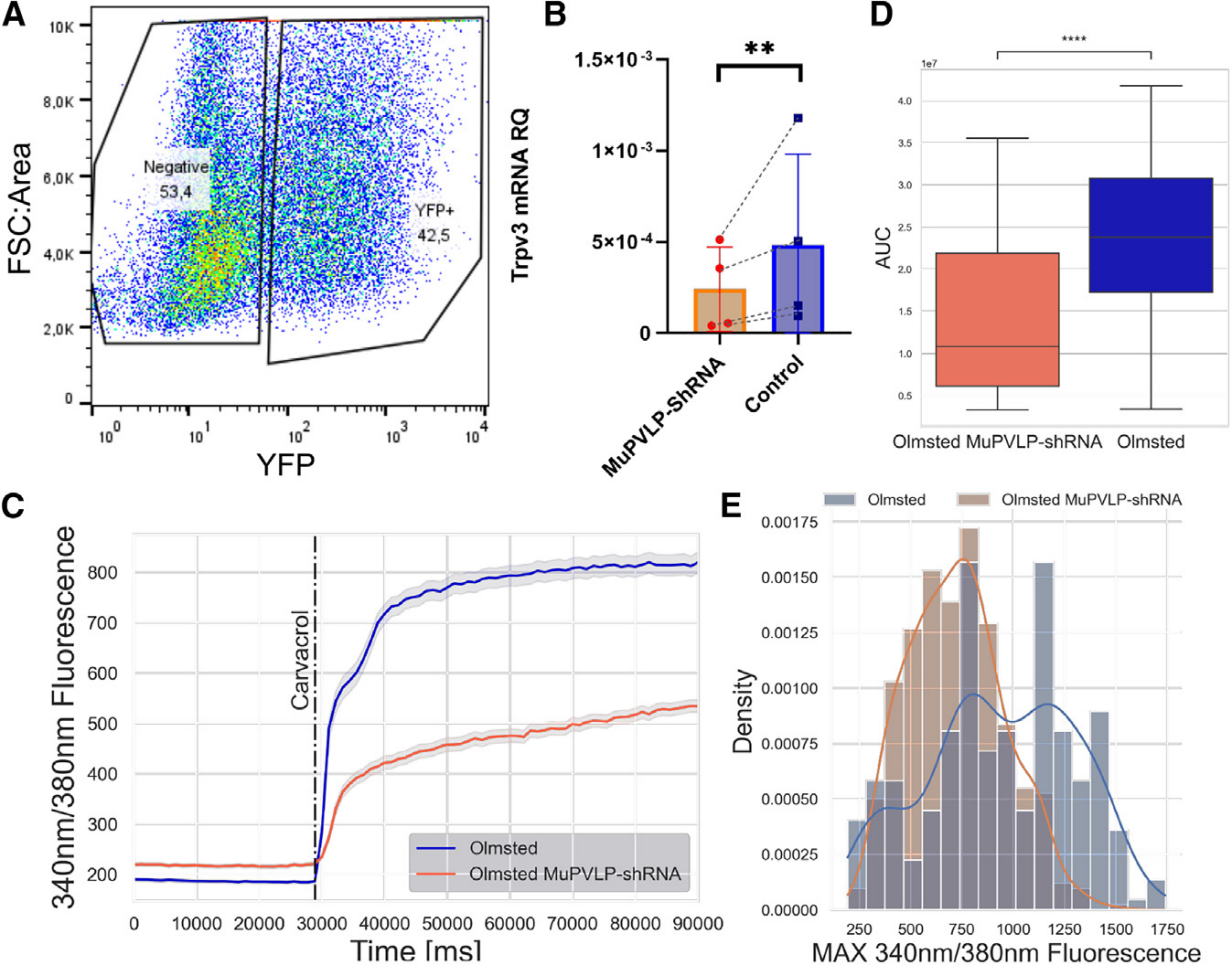
Validation of Trpv3 mRNA interference and reduced TRPV3 channel response after MuPVLP-shRNA-YFP treatment in primary keratinocytes derived from DS-Nh mice (A) FACS strategy. (B) Relative quantification of Trpv3 mRNA compared to the housekeeping gene Gapdh via qPCR. Ratio paired t test of control (YFP− primary keratinocytes, *N* = 4) vs. MuPVLP-shRNA-treated (YFP+ primary keratinocytes, *N* = 4), one-tailed *p* = 0.0065. (C) Average FURA2-AM 340/380-nm fluorescence response ±SEM to carvacrol in control (*N* = 245) and MuPVLP-shRNA-treated (*N* = 459) primary keratinocytes. (D) Mann-Whitney-Wilcoxon test with Bonferroni correction comparing the area under the curve for the first 30 s after carvacrol application between the two groups. *p* = 1.034e−30. (E) Density plot of the maximal 340/380-nm fluorescence response to carvacrol in control and MuPVLP-shRNA-treated groups.

Next, we tested the MuPVLP-shRNA-YFP on primary keratinocytes derived from DS-Nh mice. We sorted the transduced primary keratinocytes using the same approach used for KERA-308 (Figure 6A). Relative qPCR highlighted that Trpv3 transcripts were reduced to 48% ± 15% in transduced keratinocytes (Figure 6B). Functionally, MuPVLP was also able to diminish the response of primary keratinocytes to carvacrol (*p* = 1.0e−30) (Figures 6C–6E). These experiments on KERA-308 and primary keratinocytes showed the applicability of MuPVLP in delivering shRNA for silencing Trpv3 expression: the relative qPCR showed reduced levels of Trpv3 mRNA, and the calcium imaging analysis showed that cells were less responsive to the TRPV3 agonist, suggesting a reduced expression of the channel.

### Generation of PVLP-treated skin equivalents

Keratinocytes can be transduced, grown, and differentiated into a skin equivalent that can then then be transplanted to substitute a patient’s epidermis.^14^^,^^29^ To investigate PVLP applicability for autologous transgenic skin transplants, we reconstructed a skin equivalent starting from MuPVLP-treated primary keratinocytes.

We performed initial pilot experiments with MuPVLP-TdTomato, infecting skin equivalent at 7 days *in vitro*. Intriguingly, we observed that fibroblasts were preferentially targeted over keratinocytes (Figure S10). This may be because we employed rapidly dividing (and non-irradiated) NIH3T3cells. To solve this issue, we focused on pre-transducing the primary keratinocytes while alone in culture.

Editing the genome with Cas9 is a one-time event that is passed down to the cell progeny. To model this process in skin equivalents, we utilized the recombination event provoked by Cre recombinase on primary keratinocytes derived from a (Lox-STOP-Lox) LSL-TdTomato transgenic mouse. When the recombination occurs, the STOP codon in front of the TdTomato gene is excised, causing the keratinocytes and their progeny to express the fluorescent reporter. This approach thus allowed us to quantify the degree of recombination provoked by delivery of Cre by MuPVLP.

**Figure 7:**
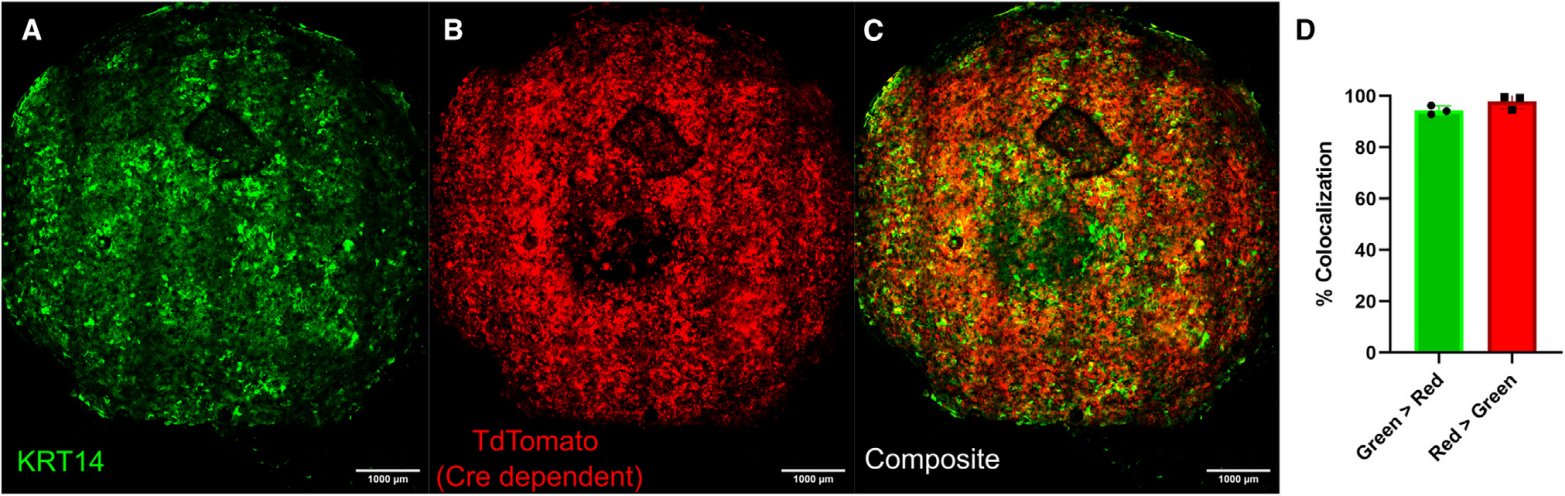
Tiled and z-stacked confocal images of skin equivalent derived from MuPVLP-Cre transduced LSL-TdTomato primary keratinocytes (A) Immunostaining for keratin14-Fluor488. (B) TdTomato expressed after recombination upon Cre transduction. (C) Composite image of the green and red channel. (D) Percentage of colocalization of green in red channel = 94.4% ± 1.4%, indicating the amount of keratinocytes (krt14 positive) that were transduced by MuPVLP-Cre; percentage of colocalization of red channel in green channel = 97.8% ± 2.2%. Skin equivalents analyzed *N* = 3.

We first isolated primary keratinocytes from an LSL-TdTomato mouse; at 1 day *in vitro*, we transduced the cells with MuPVLP-Cre and cultured them for three more days. On day 4 *in vitro*, we detached the primary keratinocytes and used them to create a skin-equivalent organotypic culture by seeding them on top of a dermal layer composed of collagen and NIH 3T3 fibroblasts. After 14 days in the appropriate medium and exposure to air, the epidermis layer was differentiated. We then fixed the samples and immunostained them for keratin14 to mark basal keratinocytes (Figures 7A–7C). Remarkably, we found that 94.4 ± 1.4% of keratin14 colocalized with the TdTomato signal (red), indicating that almost all keratinocytes had undergone recombination (Figure 7D). This indicates that PVLP-transduced keratinocytes did not face a selective disadvantage compared to untransduced cells. On the contrary, it suggests that, because PVLP preferentially transduces cells undergoing mitosis, it likely transduced the entire dividing cell population. After 20 days in culture, treated cells proliferated and came to represent nearly the entirety of the reconstructed epidermis. This phenomenon mirrors what occurs in skin equivalents generated from primary keratinocytes transduced with AAV-Cre or Lentivirus-Cre, where all the basal keratinocytes are also entirely transduced and express TdTomato (Figure S11). This indicates that MuPVLP could be employed with the same efficacy as these already accepted viral vectors.

### Rescuing Olmsted phenotype in skin equivalents

Finally, given the therapeutic potential of MuPVLP delivery of shRNAs, we investigated whether Trpv3-shRNA could mitigate the Olmsted syndrome phenotype in skin equivalents.

**Figure 8:**
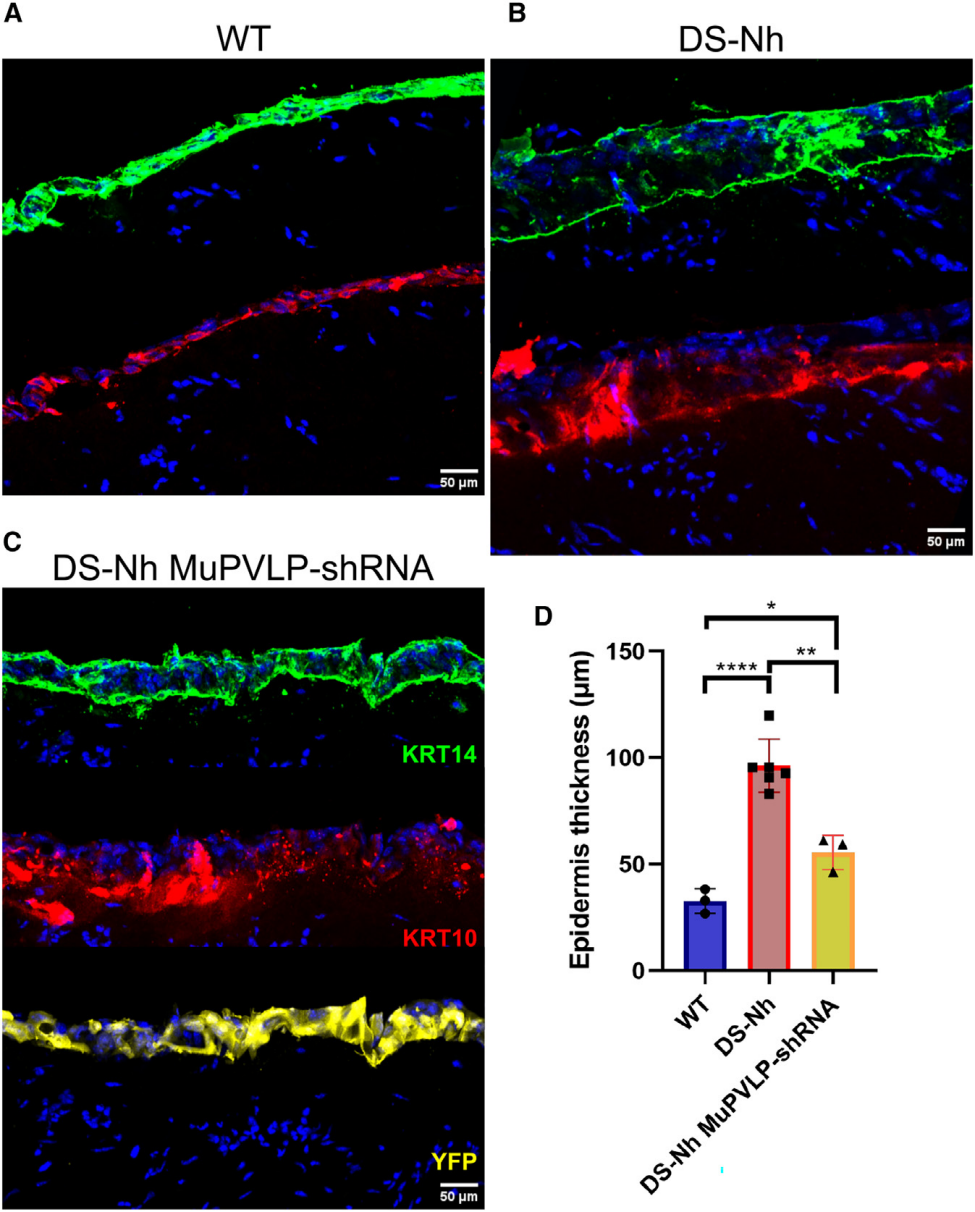
Confocal images comparison of skin equivalents Skin equivalents were derived from WT keratinocytes (A), DS-Nh keratinocytes (B), or DS-Nh keratinocytes transduced with MuPVLP-shRNA-YFP. (C) Immunostaining for DAPI (blue), keratin14 (green), and for keratin-10 (red); in yellow is the expression of YFP consequent to the transduction of MuPVLP-shRNA-YFP. (D) Measurements of the epidermis thickness across conditions. Unpaired t test WT vs. DS-Nh *p* < 0.0001; unpaired t test WT vs. DS-Nh MuPVLP-shRNA *p* = 0.0158; unpaired t test DS-Nh vs. DS-Nh MuPVLP-shRNA *p* = 0.0015.

First, we established a skin-equivalent model for Olmsted syndrome using primary keratinocytes from DS-Nh mice. Intriguingly, we were unable to observe any gross differences of the skin between samples from WT and DS-Nh mice cultured at 37°C. However, when cultured at 32°C (corresponding to the temperature in the outer skin layers,^28^ skin equivalents derived from DS-Nh mice exhibited an enlarged basal keratinocyte layer (Figures 8A, 8B, S12A, and S12B). We measured the thickness of the epidermis using antibody staining for keratin-14 and keratin-10, which are markers for the basal and suprabasal layers of the epidermis, respectively.^30^ The WT skin equivalents had an epidermal thickness of 32.7 ± 5.7 μm SD, whereas DS-Nh skin equivalents had a thickness of 96.3 ± 12.5 μm SD, almost three times thicker than the WT. This phenotype mirrored the hyperkeratosis characteristic of the syndrome *in vivo*. Our experiments also uncovered an important link between the temperature of the skin and the severity of the phenotype, perhaps due to increased activity TRPV3_G573S_ at lower temperatures (∼32°C) compared to WT TRPV3.^31^

Next, we compared skin equivalents generated from DS-Nh primary keratinocytes transduced with MuPVLP-shRNA to determine if the gene therapy could rescue the phenotype. Indeed, MuPVLP-shRNA treated skin equivalents consistently expressed YFP across the entire epidermis, indicating full transduction by MuPVLP-shRNA (Figures 8C and S12C). Although the localization of keratin 10 and keratin 14 indicated defects in maturation, MuPVLP-shRNA treatment resulted in thinner epidermis, 55.6 ± 8.0 μm SD, which was significantly lower than untreated DS-Nh (Figure 8D), suggesting a mitigation of the hyperkeratosis associated with Olmsted syndrome phenotype. While the treated skin equivalents are still thicker than the WT, the reduction to about half of the untreated DS-Nh thickness demonstrates the potential efficacy of MuPVLP in delivering an *ex vivo* gene therapy to a skin equivalent.

## Discussion

In this study, we employed PVLPs to deliver a variety of DNA constructs encoding fluorophores, Cre recombinase, GCaMP8s, SaCas9, and shRNA to keratinocytes to demonstrate its applicability for gene therapy. We provide evidence that PVLP can be employed to efficiently transduce primary keratinocytes for *ex vivo* gene therapy, with substantial advantages over other viral vectors such as AAV and Lentivirus. We found that SaCas9 and shRNA delivered via PVLP could disrupt the *Trpv3* gene or reduce Trpv3 expression. Both strategies led to a reduction of the channel activity, abolishing the hyperactivity that underlies Olmsted syndrome. We generated skin equivalents starting from PVLP-treated primary keratinocytes and demonstrated that the epidermis was entirely composed of PVLP-transduced keratinocytes. Furthermore, MuPVLP-shRNA treatment significantly reduced the hyperkeratosis in skin equivalents derived from DS-Nh mice, suggesting a partial rescue of the Olmsted syndrome phenotype. These findings indicate that PVLP could be a powerful tool for *ex vivo* skin gene therapy.

We focused on Olmsted syndrome as a proof of concept to determine the efficacy of PVLPs for gene therapy, particularly for gain-of-function genodermatosis. Olmsted syndrome is caused by a single point mutation in *Trpv3*, recapitulated by Gly573Ser in the DS-Nh mouse model.^26^ While it is unlikely that disruption of the entire *Trpv3* gene utilizing *ex vivo* gene therapy (as performed here) would be a suitable therapeutic option, we utilized this knockout strategy as a means of demonstrating the potential of PVLPs in delivering gene therapy. One could argue that the knockout phenotype would still be preferable to the Olmsted gain-of-function phenotype: *Trpv3* knockout mice have innocuous hair abnormalities (wavy fur) and only present defects in keratinocyte maturation and a more permissive skin barrier during fetal development and not post birth.^7^^,^^32^^,^^33^ However, more precise genome-editing techniques, such as adenine base editors (ABEs), prime editing, or epigenome editing should be considered to develop a therapy to correct Olmsted mutation.^34^^,^^35^^,^^36^ These may be especially valid given the large packaging capacity of PVLP. It should also be pointed out that other therapeutic options exist for Olmsted syndrome, in particular the recent discovery that topical application of the EGFR inhibitor erlotinib shows remarkable efficacy in managing Olmsted symptoms such as hyperkeratosis and pain, which can resolve within 3 months from the start of the therapy.^37^ Olmsted patients need to be maintained on erlotinib treatment to stay in remission, as long as no considerable adverse effects are observed. Gene therapy now presents the potential for a definitive cure at the source of the monogenic disease, making it a promising avenue for further exploration.

Beyond Olmsted syndrome, we propose a general workflow utilizing cell-free and cell-assembled PVLPs. Cell-free-assembled PVLPs are particularly suited for rapidly testing a library of constructs, as they can co-assemble around any DNA fragment smaller than 8 kb. This method is especially advantageous for identifying the most effective gRNA or siRNA in a difficult-to-transfect cell line (such as KERA-308), offering higher efficiency than Lipofectamine transfection while eliminating the need for time-consuming plasmid cloning to incorporate the construct into a viral vector-compatible plasmid. Once the best candidate is identified, the SV40 promoter can be cloned into the plasmid of interest, which is essential for efficient production of cell-assembled PVLPs.^11^ To effectively transduce primary keratinocytes and presumably other primary cells, PVLPs must be cell assembled, as cell-free assembly reaction likely lacks some factor necessary for proper encapsidation. Nevertheless, since many mammalian expression plasmids already contain an SV40 promoter, cell-assembled PVLPs can be generated rapidly and easily.

We focused our efforts on using PVLP for *ex vivo* gene therapy because Papillomavirus is known to be highly immunogenic *in vivo*. Indeed, most *in vivo* studies with PVLPs have been conducted on immunosuppressed animals,^38^^,^^39^ and PVLPs have been proposed as noninfectious viral vaccines due to their strong immunity induction.^40^ In a pilot *in vivo* experiment, topically applied PVLP provoked limited transduction of keratinocytes, with higher transduction levels in fibroblasts and putative Langerhans cells. Consequently, we shifted our focus to organotypic skin cultures. The immunogenicity of viral vectors is a constant challenge for *in vivo* gene therapy. To effectively utilize PVLPs *in vivo*, serotype selection or capsid engineering may be necessary to enable them to evade the immune response.

However, as demonstrated here, PVLP has proved to be very effective for *ex vivo* gene therapy of the skin and it could be employed as an alternative to retroviruses, which were already employed for *ex vivo* gene therapy of a human case of severe epidermolysis bullosa.^14^ In our study, we successfully generated skin equivalents that exhibited nearly complete recombination following Cre recombinase delivery. We hypothesize that PVLPs may preferentially transduce holoclones and meroclones—the primary keratinocytes with the capacity for proliferation and self-renewal, from which the skin equivalent is formed.^41^ Alternatively, PVLPs may effectively target all keratinocyte clones (holoclones, meroclones, and paraclones), with only holoclone- and meroclone-derived keratinocytes persisting after multiple passages. A clonal analysis is necessary to determine which of these scenarios is correct. This preferential targeting could be due to PVLP’s ability to transduce only actively dividing cells.^21^^,^^22^ While this characteristic may limit PVLP’s use in transducing non-dividing cells like neurons, it can also be seen as an advantage, as it preferentially targets stem cells and highly dividing cells. For this reason, PVLPs were already proposed for targeting cancer cells.^42^^,^^43^^,^^44^

In this study, we proposed Papillomavirus as a new viral vector for skin gene therapy. Papillomavirus offers several advantages as a vector: it can encapsidate up to 8 kb of genetic material, allowing it to deliver larger constructs like base editors or large Cas9 proteins that exceed the 4.7-kb packaging limit of AAVs. Additionally, unlike retroviruses, Papillomavirus does not integrate its cargo into the host genome, thus reducing the risk of unintended off-target effects and minimizing the possibility of disrupting host genes.

Until now, *ex vivo* gene therapy has predominantly relied on retroviral vectors or the nucleoporation of CRISPR-Cas9 ribonucleoproteins (RNPs).^14^^,^^45^^,^^46^ PVLPs can be considered direct competitors to NILVs, as both share a similar cargo capacity and remain episomal. However, NILVs are known to exhibit lower expression levels compared to their integration-competent counterparts.^20^ In contrast, our experiments demonstrated that PVLPs achieved transduction efficiencies in primary keratinocytes that were superior to those of integrating lentiviruses. A widely used method for delivering CRISPR-Cas9 *ex vivo* is the nucleoporation of RNP complexes. While effective, nucleoporation is associated with several limitations, including high cell mortality, challenging optimization processes, and the requirement for large cell numbers.^47^ Moreover, nucleoporation is inherently restricted *ex vivo* and cannot be applied for *in vivo* therapy. However, the possibility of delivering RNA or protein instead of DNA for enhancing safety and not having integration or overexpression issues is very appealing; for this reason, several new engineered viral-like particles such as such as Lentiflash particles, Nanoblades, or eVLPs, are under development for the delivery of Cas-gRNA in the form of RNPs or as non-integrative RNA.^48^^,^^49^^,^^50^ Meanwhile, non-viral delivery methods, such as lipid nanoparticles (LNPs), are also advancing. Although LNPs are less efficient at transducing cells compared to viral vectors, they do offer the potential for transient expression of a gene of interest. Notably, some studies have successfully utilized LNPs for *ex vivo* gene therapy in 3D skin models^51^ and achieved efficient *in situ* gene delivery via LNPs for human skin therapy.^52^

Expanding our repertoire of viral vectors is important, as it equips us with new tools to address different challenges in different contexts. For instance, Gurevich et al.^53^ successfully utilized a herpes simplex-based viral vector for *in vivo* gene therapy of epidermolysis bullosa. PVLP holds significant potential for *in vivo* gene therapy. With appropriate engineering to mitigate its immunogenicity, PVLP could overcome these limitations. Its episomal nature, combined with its ability to achieve higher transduction efficiencies than lentiviruses in primary keratinocytes, positions PVLP as a safe and competitive alternative for therapeutic use, particularly for delivering CRISPR-Cas-based technology. To fully establish PVLPs as viable gene therapy vectors, future research should focus on evaluating their long-term expression levels, *in vivo* immunogenicity, and translational potential for clinical applications.

## Materials and methods

### Animals

All experiments with WT C57BL/6J, DS-Nh, LSL-TdTomato mice were performed by the European Union (EU) guidelines (2010/63/UE) and Italian law (Decree 26/14) and were approved by the local authority veterinary service and by SISSA animal well-being committee (OPBA). All efforts were made to minimize animal suffering and to reduce the number of animals used. Animal use was approved by the Italian Ministry of Health (nos. 22DAB16, 22DAB.N.HMP, and 22DAB.N.9FV) in agreement with EU Recommendation 2007/526/CE.

### Cell lines

Keratinocyte KERA-308 and fibroblast NIH/3T3 (ATCC) murine cell lines were cultured in DMEM Glutamax (Gibco, 31966047) + 10% FBS (Euroclone ECS0180L) + 1% penicillin/streptomycin (penstrep) (Euroclone ECB3001D). HEK293TT (ATCC) cells were maintained in DMEM Glutamax supplemented with 10% FBS, 1% penstrep, 1% MEM non-essential amino acid (Euroclone ECB3054D), 1% L-glutamine (Euroclone ECB3004D), and 250 μg/mL hygromycin B (Thermo Fisher Scientific, 10687-010). Trypsinization of cells for passaging was performed by incubation with 0.05% Trypsin-EDTA solution (Sigma, T4174) for 3–15 min depending on the cell type.

### Primary keratinocyte culture

Primary keratinocytes were isolated from adult mice following the protocol described by Li et al.^54^ Briefly, mice were sacrificed using CO_2_, and skins from tails and paws were dissected and digested overnight in a solution of dispase II at 4 mg/mL (Sigma, D4693) dissolved in defined keratinocyte-serum free medium (1×) (KSFM; Gibco, 10744-019) containing 1% penicillin/streptomycin antibiotic and 1 mL of supplied growth supplements. The next day, the epidermis was separated from the dermis and further digested for 20 min at room temperature (RT) in TrypLE Express solution (Gibco, 12604013). After digestion, the epidermal sheets were vigorously rubbed on a Petri dish with KSFM solution to allow the release of single cells. Cells were filtered through 100-μm cell strainers (Falcon), centrifuged (4°C, 1,000 rpm, 5 min), and plated at high confluency on the desired well plate or flask coated with rat-tail collagen-I coating solution (Sigma, 122-20). Cells were cultivated in supplemented KSFM in a humidified 37°C cell incubator with 5% CO_2_ and medium was changed every 2–3 days.

### Mouse skin equivalents

Skin equivalents were produced following the protocol published by Merck (https://www.sigmaaldrich.com/IT/it/technical-documents/protocol/cell-culture-and-cell-culture-analysis/3d-cell-culture/organotypic-epidermal-skin-culture) with some adjustments. For each equivalent, we prepared the dermal layer by mixing on ice 90 μL of rat-tail collagen (Sigma, 08-115), 24 μL of 5× reconstitution buffer (1.1% NaHCO_3_, 0.025 N NaOH, 100 mM HEPES, 5× DMEM/F12), and 6 μL of NIH3T3cells (4 × 10^6^ cells/mL cell suspension). The collagen/fibroblast mixture was applied directly to the center of each Millicell 24-well hanging cell culture insert (Millipore, PTHT24H48) and incubated at 37°C for 30 min to allow the collagen to gelatinize. It was then equilibrated with DMEM until the primary keratinocytes were ready. 150 μL of cultured primary keratinocytes (8 × 10^5^ cells/mL) were applied over each collagen/fibroblast layer and 600 μL of supplemented KSFM to the outside of the inserts. The following day, an additional 150 μL of keratinocyte medium was added to the inside of each insert and incubated at 37°C for two more days. On the fourth day, the medium was aspirated from the outside of each insert and the medium changed to 500 μL of 3dGRO Skin Differentiation Medium (Sigma, SCM310); this quantity allows the skin culture to be maintained at the air:liquid interface. The skin cultures were incubated at 32°C or 37°C for 10 more days, changing the medium every other day.

### PVLP production

PVLPs were made following the Center for Cancer Research protocol (https://ccrod.cancer.gov/confluence/display/LCOTF/PseudovirusProduction).^11^^,^^55^ HEK293TT at 50%–60% confluency in a T75 flask were transfected with 20 μg of the capsid plasmid—p16sheLL (Addgene, 37320) or pMusheLL (Addgene, 47023)—and 20 μg of the desired cargo plasmid (which must contain an SV40 promoter and be smaller than 8 kb) by condensation with linear polyethyleneimine (PEI). As cargo plasmids we employed ptwB (Addgene, 48735), pCMV-Cre (Addgene, 123133), pGP-CMV-jGCaMP8s (Addgene, 162371), and customized pX601-mCherry (Addgene, 84039) and AAV-shRNA_Tet3 (Addgene, 85740). 48 h post transfection, producer cells were collected by trypsinization together with the medium, the cells were spun down, and the supernatant was discarded. The pellet was moved into a low-binding Eppendorf; resuspended with 1.5 pellet volumes of DPBS, 1% penstrep, 10 mM MgCl_2_; then mixed with 0.5% Triton X-100, 40 mM NaPO_4_ and 1,000 units/mL of salt-active nucleases (Sigma, SRE0015). The mixture was incubated overnight at 37°C to allow the correct maturation of the capsid. The following day, the NaCl concentration was brought to 850 mM and incubated on ice for 10 min. The salt lysate was clarified by spinning for 5 min at 5,000 × *g* and the supernatant was transferred into a new low-binding tube. The pellet material was re-extracted by resuspending into one pellet volume of DPBS/0.8 mM NaCl and the second clarified solution was added to the first. The pooled solution was re-clarified at 5,000 × g for 5 min. PVLP was enriched via Sepharose spin column (Pierce centrifuge columns, Thermo Fisher Scientific, 89896; Sepharose B, Sigma, 4B200): 2 mL of column bed volume of Sepharose was equilibrated and blocked with DPBS, 0.8 M NaCl, and 0.1% bovine serum albumin (BSA) solution. The suspension medium was then exchanged to DPBS/0.8M NaCl/0.01% Pluronic without BSA. The sample was loaded on the dry column and the eluate was collected, aliquoted, and snap-frozen for subsequent use. PVLPs were quantified by absolute qPCR adjusting the Addgene protocol for AAV titration (https://www.addgene.org/protocols/aav-titration-qpcr-using-sybr-green-technology/).^56^ The cargo plasmid with *ad hoc* primers was employed to generate the standard curve.

### Cell-free-assembled PVLP

Empty capsids were purified as above from HEK293TT cells transfected with 40 μg of the capsid plasmid. The cell-free assembly conditions were based on Cerqueira et al.^13^ The reaction was prepared by mixing 5 μg of linearized plasmid with 1 μg of empty capsid (based on L1 amounts quantified by Coomassie staining) in a citrate buffer at pH 5.2 with 0.002% Tween-80. The reaction was incubated for 48 h at 37°C.

### AAV production

Recombinant AAV1/2 carrying Cre as cargo was produced in HEK293T cells as described previously.^57^ Cells were harvested 3 days post transfection, lysed with Triton X-100 at 0.5%, nuclease treated, concentrated by tangential flow filtration, and purified using isopycnic ultracentrifugation. Vector genome titration was performed using qPCR with primers targeting the ITR region of the viral cargo.

### Lentivirus production

Lentiviral particles were prepared by transfecting HEK293T in a 10-cm dish with 10 μg of Cre-IRES-PuroR cargo plasmid (Addgene, 30205) together with packaging plasmids: 2.5 μg of pMD2-VSVG (Addgene, 12259) and 7.5 μg of psPAX2 (Addgene, 12260) by using linear PEI. After 8 h, the HEK293 medium was changed. 24 and 48 h post transfection, supernatant was collected and passed through a 0.45-μm filter. The collected medium was centrifuged at 50,000g for 2 h 30 min at 20°C in polyallomer bottles (Beckman 357003). The supernatant was discarded and the pellet resuspended in 20 μL of PBS every 10 mL of starting medium, aliquoted, snap-frozen, and stored at −80°C. Lentivirus infectious titer was measured by flow cytometry: 2 × 10^5^ cells/well LSL-TdTomato HEK293T were seeded in a 24-well plate and immediately infected with a 3-fold serial dilution of the Lentivirus-Cre. 48 h after transduction, cells were collected and analyzed by flow cytometry for TdTomato expression. The transfecting unit (TU)/mL was calculated assuming that 1 viral particle per 1 cell condition happened when the transduced fluorescent cells were in the 10%–18% range.

### Flow cytometry for viral vector comparisons

HEK-293T and KERA-308 in a 24-well plate were either transfected with Lipofectamine 3000 Transfection Reagent (Invitrogen, L3000008) according to manufacturer’s instructions, or with 5,000 MOI of cell-assembled PVLP (human or murine), or with cell-free-assembled PVLP employed to encapsidate 1 μg of cargo DNA per well. After 3 days, cells were imaged at an inverted microscope or were detached, resuspended in FACS buffer (1× PBS; 5% FBS), and counted via flow cytometry at an S3e Cell Sorter (Bio-Rad). LSL-TdTomato primary keratinocytes were transduced with different MOIs of AAV1/2-Cre, Lentivirus-Cre with 8 μg/mL polybrene, or MuPVLP-Cre and were analyzed at 5 or 10 days post infection (dpi). The medium was changed the day after in the case of AAV and MuPVLP, whereas it was changed after 4 h in the case of Lentivirus to avoid excessive cytotoxicity. The keratinocytes were dissociated with trypsin for 15 min, resuspended, and incubated for 30 min in PBS containing eFluor450 viability dye (Invitrogen, 65-0863) according to the manufacturer’s instructions. After incubation, cells were suspended in FACS buffer. Transduced cells were counted via flow cytometry. The results were analyzed with FlowJo_v10.10.0.

### SaCas9 plasmid handling and cloning strategy

Three gRNA candidates for Trpv3 gene disruption were evaluated by cloning them into pX601 plasmid with a hygromycin resistance cassette. gRNA-KO-2: 5′-TCCTGGACAGGTTCATCAACG-3′; gRNA-KO-3: 5′-CTCACCAATGTAGACACAACG-3′; gRNA-KO-4: 5′-GTGTCTACATTGGTGAGGTCA-3′. Once the best gRNA (gRNA-KO-4) was selected, it was cloned into the plasmid pX601-mCherry (Addgene, 84039) encoding for the SaCas9 via ligation after the double cutting with BbsI-HF (NEB, R3539). pX601-mCherry-Trpv3gRNA plasmid was too large (8,123 bp) to be directly used for producing PVLPs. For this reason, it was digested with XhoI (R0146, NEB) and NotI (R0189, NEB) restriction enzymes for 16 h in NEBuffer r3.1. The digestion mix was separated by electrophoretic run in a 0.8% agarose gel, and the 5,236-bp band was excised and purified via NucleoSpin Gel and PCR Clean-up kit (Macherey-Nagel, FC140609N). 5 μg of the digested plasmid was subsequently used for cell-free-assembled PVLP preparation. To produce cell-assembled PVLP, we reduced the size of the plasmid by replacing the larger CMV promoter (584 bp) with the SV40 promoter (330 bp), also necessary for PVLP production. To further shrink the size of the plasmid from 7,869 to 7,117 bp, the mCherry reporter was removed by digesting the plasmid with BamHI-HF (R3136, NEB) and SacI-HF (R3156, NEB). The insert was designed to have the same BamHI and SacI restriction sites and to contain another SV40 nuclear localization sequence (NLS) and a STOP codon (Figure S13). The insert was generated by annealing 10 μ L of the 100 μM forward oligo gatccccaaagaagaagcggaaggtctaagagct with 10 μ L of the 100 μM reverse oligo cttagaccttccgcttcttctttggg in 80 μ L of an annealing buffer (10 mM Tris, 60 mM NaCl, 1 mM EDTA, pH 7.5–8.0). The annealing mix was heated to 95°C for 3 min and allowed to cool at RT for at least 30 min to let the two oligos hybridize.

### Trpv3 gene disruption via SaCas9

KERA-308 cells were transduced with 5,000 MOI of MuPVLP-SaCas9 and, 7 days post treatment, genomic DNA was extracted using the DNeasy Blood & Tissue Kits (Qiagen, 69504), according to the manufacturer’s instructions. PCR amplicons of on-target site were amplified via Trpv3 exon 9 primers (forward AGCTGATGGTTTGGCTCTCT and reverse CCCTCTATGCCAGACACCAT) purified with NucleoSpin Gel and PCR Clean-up kit (Macherey-Nagel, FC140609N). Sanger traces were generated by Eurofins Genomics with Mix2Seq kit and analyzed with the TIDE web tool (http://tide.nki.nl)^58^ for revealing indel generation. Default parameters were used for the analysis. The same workflow was employed for primary keratinocytes. Western blot was performed on MuPLVP-SaCas9-treated KERA-308 14 dpi. Cells were lysed in RIPA buffer, and protein content was quantified via Pierce BCA Protein Assay Kit (Thermo Fisher, A55864). Denaturalized proteins were run on 12% Mini-PROTEAN TGX Precast Protein Gel (Bio-Rad, 4561043), transferred to the membrane, and incubated with TRPV3 Polyclonal Antibody (Invitrogen, PA5-11471) and GAPDH Monoclonal Antibody (GA1R) (Invitrogen, MA5-15738).

### Gene silencing via shRNA

Four shRNA candidates—GCTGGAAATCATCGTCTACAA (1); ACACACGUGUCCUUCCUUA (2); UCAUCGUCUACAACACCAA (3); CACACGUGUCCUUCCUUAA (4)—were generated using the siRNA Wizard Online Tool (https://www.invivogen.com/sirna-wizard). We employed TCAAGAG as loop sequence between the sense and the antisense part of the shRNA. Oligos encoding for shRNAs were synthesized by Sigma Aldrich, with overhangs for BamHI and XbaI. shRNA sequences were cloned into AAV-shRNA_tet3 plasmid (Addgene 85740), which has a YFP reporter. BamHI (NEB 3136) and XbaI (NEB R0145) restriction enzymes were employed to digest AAV-shRNA_tet3, and the oligos were ligated using T4 DNA Ligase (NEB, M0202). KERA-308 cells were transduced with the candidate plasmids and sorted 5 days post treatment based on YFP expression using an S3e Cell Sorter (Bio-Rad). RNA was extracted from both the YFP+ and YFP− (control) populations with RNeasy Protect Mini Kit (Qiagen, 74124) and converted to cDNA with PrimeScript RT Reagent Kit with gDNA Eraser (Takara, RR047). The efficiency of the interference of the candidate shRNA was evaluated via relative qPCR normalizing on housekeeping gene Gapdh and comparing the YFP+ to the YFP− population. Primers for the relative qPCR were Trpv3 forward, CAGCAGAACTCCACCTACCC; Trpv3 reverse, TTGAGGAGGAGGACGAAGGT; Gapdh forward, GAAGGGCTCATGACCACAGT; Gapdh reverse TGCAGGGATGATGTTCTGGG. The same workflow was employed for primary keratinocytes. For producing cell-assembled PVLPs, the shRNA plasmid lacked the SV40 promoter and origin; thus, we cloned inside the backbone of pCMV-Cre plasmid the sequence of interest by using MfeI and XhoI restriction enzymes.

### Immunofluorescence

Cell culture of primary keratinocytes treated with MuPVLP-SaCas9 at 7 dpi were fixed with 4% PFA for 5min, permeabilized with Triton X-0.1% for 10 min at 37°C, blocked in 3% BSA for 30 min at RT, incubated with SaCas9 Monoclonal Antibody (11C12) (Thermo Fisher, A01951-40) in 0.1% BSA for 2 h at RT, washed, incubated with secondary antibody, incubated with DAPI, and mounted. Skin equivalents were fixed in 2% PFA for 1 h at 4°C and either used as whole mounts or embedded in Killik O.C.T. (Bio-Optica, 05 9801) and cut at 30 μm at the cryostat. The fixed samples were washed; blocked in 3% goat serum (Sigma, G9023), 0.01% Tween 20, and PBS solution for 1 h; and incubated with primary antibodies in blocking solution overnight at 4°C. The next morning after washing, secondary antibodies in the blocking solution were added and incubated for 2 h at RT. DAPI (Sigma, 32670) was added during the first wash and left for 20 min. After two more washes with PBS, stained skin equivalents were mounted with Mowiol 4-88 (Sigma, 81381). For immunofluorescence experiments, rabbit anti-keratin14 (1:1,000) (BioLegend, 905301) and mouse anti-cytokeratin10 (1:500) (Thermo Fisher, MA1-06319) primary antibodies were used. All secondary antibodies were Alexa conjugated and were used at a concentration of 1:1,000. Images were acquired using a Leica SP5 confocal microscope and analyzed using ImageJ. Colocalization was quantified with Coloc2 plugin.

### Calcium imaging

KERA-308 or primary keratinocytes seeded on glass were incubated with 3 μM Fura-2 AM (Sigma, 47989) and 2.5 mM Probenecid (Sigma, P8761) at 37°C for 40 min and washed and imaged in a calcium imaging buffer (150 mM NaCl, 5 mM KCl, 1.8 mM CaCl_2_, 1.2 mM MgCl_2_, 25 mM HEPES, and 10 mM D-glucose, pH 7.4). Fluorescence microscopy was performed on an Axiovert 135 inverted microscope (Zeiss, Germany) using a Plan - NEOFLUAR 20×/0.5 NA objective, Furaled (Crisel) with 340 nm and 385 light-emitting diodes (LEDs) as light source, and a Till Image-QE CCD Camera (Zeiss) for recording. Before the start of every recording, 50 μM TRPA1 antagonist HC-030031 (Tocris, 2896) was added to the cells. To stimulate the calcium influx due to TRPV3 opening, we added 500 μM carvacrol (Sigma, 282197). TILLvisION software (Zeiss) was used to record image data. After background (B340, B380) subtraction in each channel (F340, F380), the ratio (R) of fluorescence elicited by two excitation lights was calculated as R= (F340 – B340)/(F380 – B380). Data were analyzed in TILLvisION and on Python 3.

### Statistical analysis

All statistical data are presented as SEM along with the number of samples analyzed (*N*). Student t test was performed on GraphPad Prism 8.3.0 for the analysis of the silencing effect of shRNA and for the thickness of the epidermis. Mann-Whitney-Wilcoxon test two-sided with Bonferroni correction statistical analysis of the area under the curve was employed to analyze the significance of the calcium imaging traces. Statistical significance was assumed at *p* = 0.05.

### Conclusions

PVLPs represent a promising method for delivering CRISPR-Cas-based technology. PVLPs can encapsulate larger DNA segments compared to AAVs, which are often too small to accommodate most Cas9 and base editors. The DNA delivered by PVLPs remains episomal, providing an advantage over lentiviruses by preventing the editing machinery from integrating into the genome. This avoids continuous expression and reduces the risk of off-target effects. As proof of concept, we applied PVLP for the gene therapy of Olmsted syndrome. We demonstrate efficient targeting of primary keratinocytes by PVLP and the ability to silence or disrupt the *Trpv3* gene. Our results showed that treatment with PVLP-shRNA partially rescued the Olmsted syndrome phenotype in skin equivalents. Thus, we propose PVLP as a promising alternative vector for *ex vivo* gene therapy of the skin.

## Data availability

The data presented in this study are available on request from the corresponding author.

## Acknowledgments

This research was funded by support from 10.13039/100013104SISSA and 10.13039/100013060EMBL. We thank Massimo Righi, Micaela Grandolfo, and SISSA technical staff for support. Data and materials are available from the authors under a data or material transfer agreement. “Channel-lid-ions-membrane” and “Plasmid” icons by Servier https://smart.servier.com/are licensed under CC-BY 3.0 Unported https://creativecommons.org/licenses/by/3.0/.

## Author contributions

F.D., P.A.H., and J.A.H. conceived the study. F.D. performed the experiments with help from J.D., E.M., M.R., and F.C.R. F.D. and P.A.H. wrote the manuscript with feedback from all authors.

## Declaration of interests

The authors declare no competing interests.

## Supplemental information

Document S1. Figures S1–S13

Document S2. Article plus supplemental information
