# Supplementary material for: Papillomavirus-like particles as vectors for ex vivo gene therapy of the skin
Source: Mol Ther Nucleic Acids. 2025 Mar 5;36(2):102501. doi: 10.1016/j.omtn.2025.102501 (PMC11960642; doi:10.1016/j.omtn.2025.102501)
Supplement: Document S1. Figures S1–S13 [file mmc1.pdf]

OMTN, Volume *36*

Supplemental information

Papillomavirus-like particles as vectors

for *ex vivo* gene therapy of the skin

Francesco Diversi, Juliette Dabin, Elisa Mazza, Mirko Rinaldin, Fernanda de Castro Reis, Jamie A. Hackett, and Paul A. Heppenstall

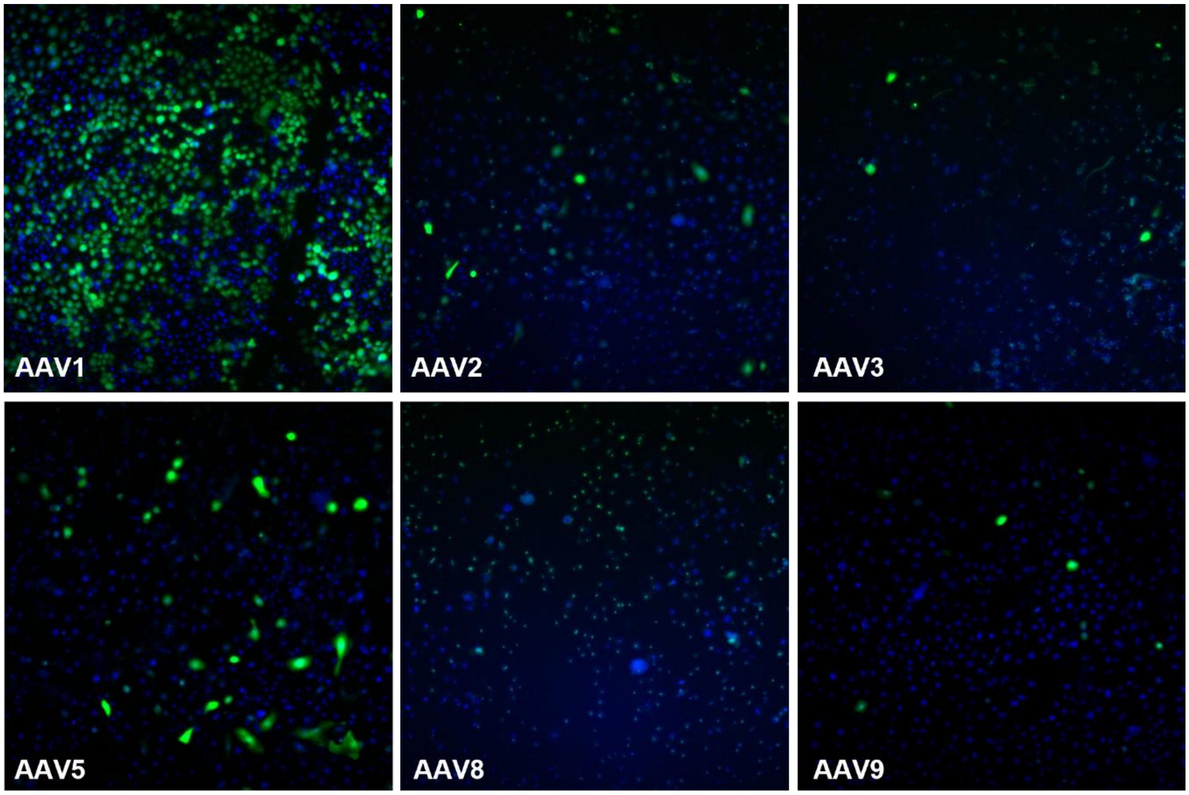

Figure S1. The fluorescent reporter GFP was employed to evaluate the transduction efficiency of different AAV serotypes at 10^5 MOI in primary murine keratinocytes at 5 days post-infection. In blue nuclei highlighted via Hoechst staining.

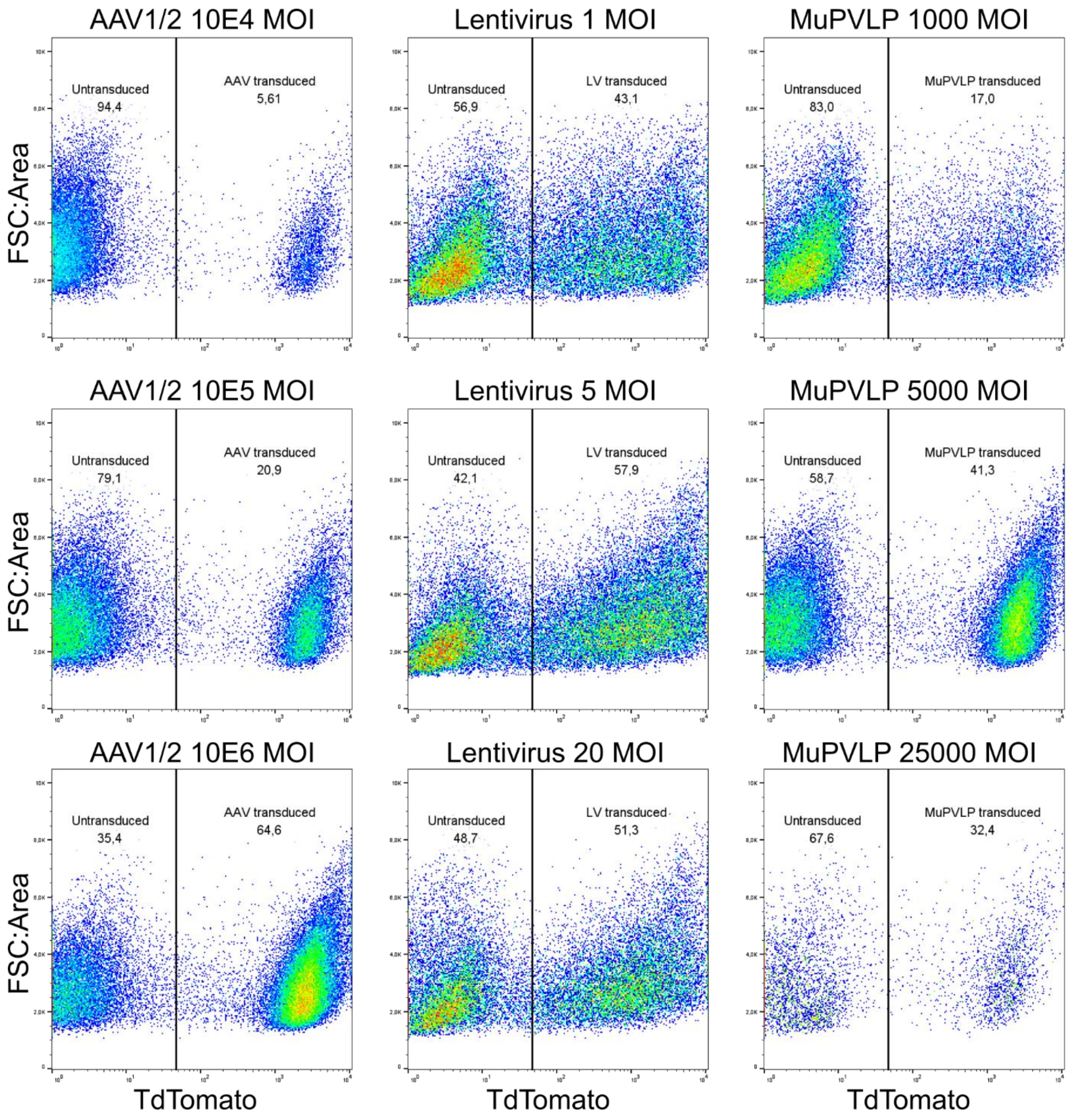

Figure S2. The efficiency of transduction of MuPVLP, Lentivirus, and AAV was quantified at different Multiplicity of Infection in the range typical for each vector, 5 days post-infection, via TdTomato expression dependent on Cre recombination.

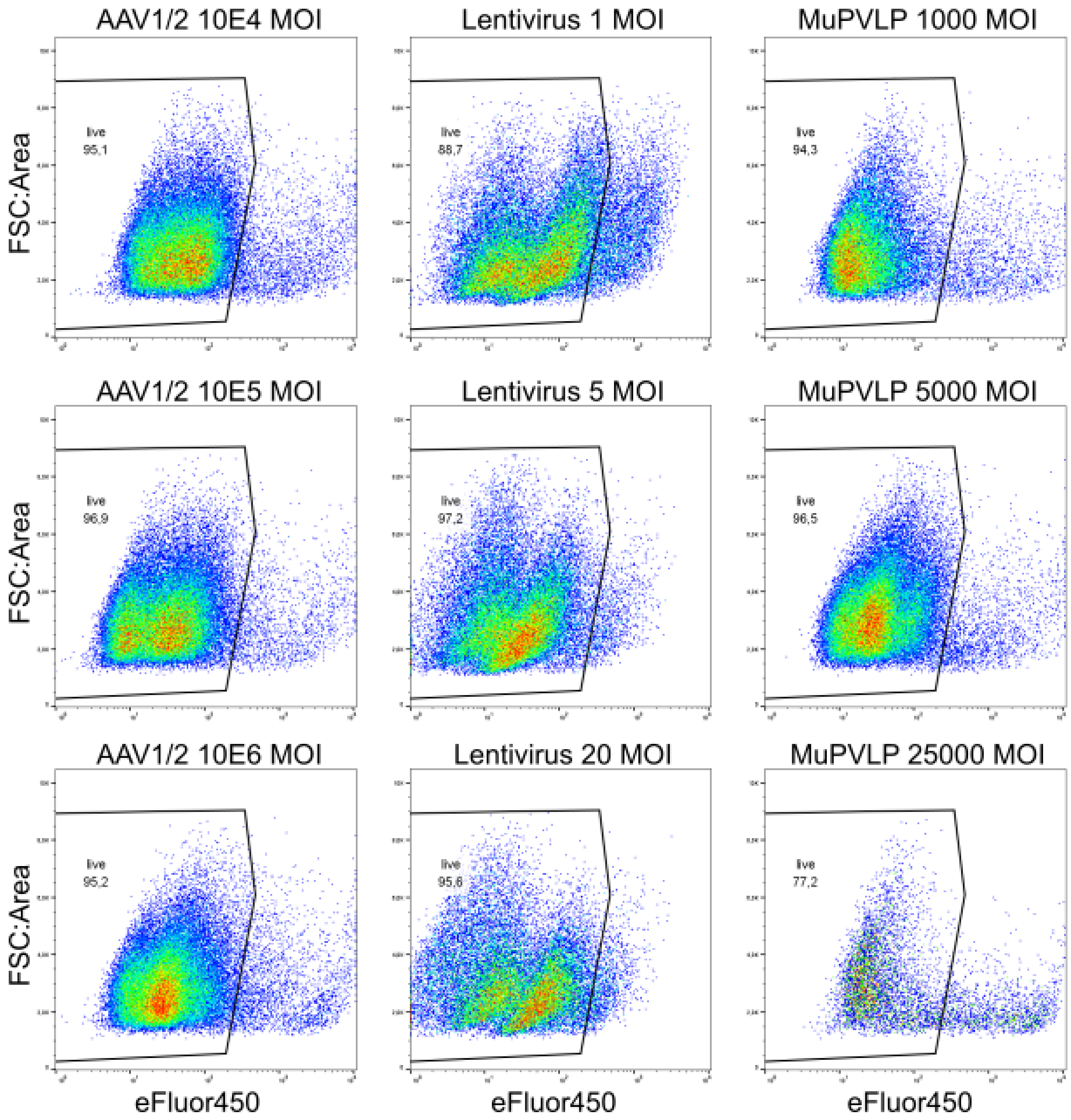

Figure S3. The cytotoxicity of MuPVLP, Lentivirus, and AAV was quantified at different Multiplicity of Infection, 5 days post-infection, via eFluor450 incorporation.

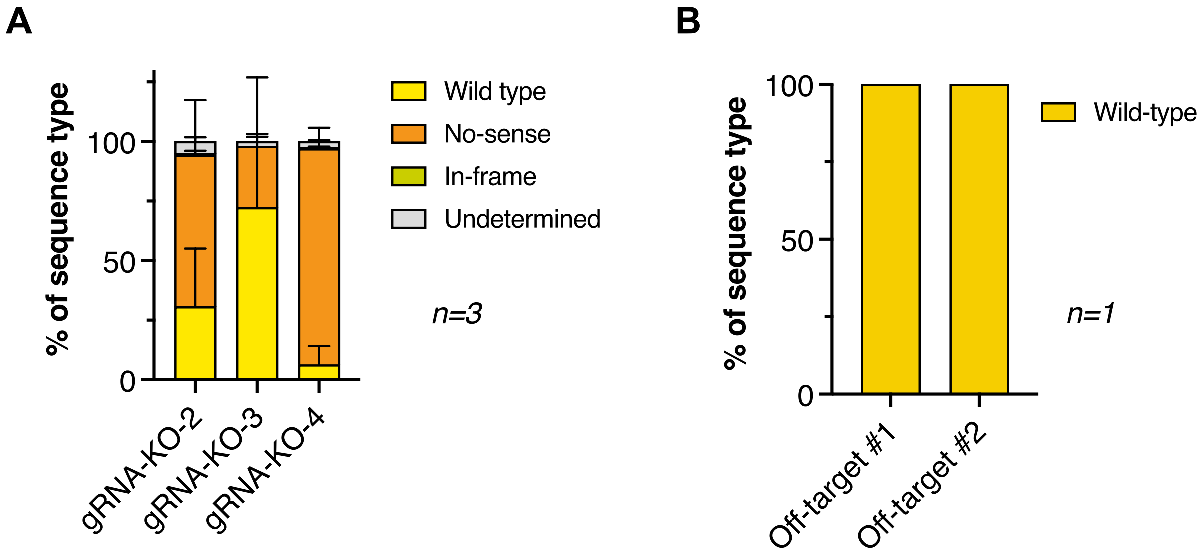

Figure S4. (A) We evaluated three candidate gRNA against the Trpv3 gene in the KERA-308 cell line. The cells were transduced with plasmids encoding for SaCas9, the candidate gRNA, and a hygromycin resistance cassette. Cells were cultivated for 3 weeks under hygromycin selection, then genomic DNA was extracted, and the targeted locus sequenced. We chose gRNA-KO-4 to perform all the experiments with MuPVLP. (B) The two top off-targets for gRNA-KO-4: Off-target #1: TGTCTACTTTGATGAGGCAAA; Off-target #2: TGCTGTTCATTGGTGAGGTCA; were sequenced for indel detection. Neither of the two top off-targets showed any disruption.

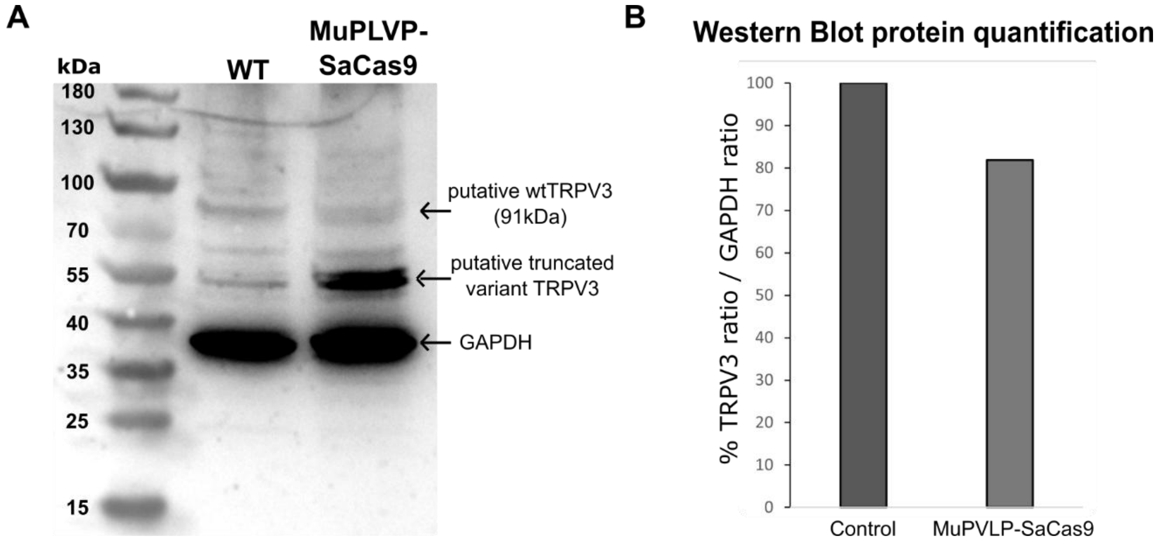

Figure S5. Western Blot for quantifying TRPV3 protein in MuPVLP-SaCas9 treated and control (WT) KERA-308. (A) Western Blot membrane, with arrows pointing to the putative wtTRPV3 band (expected at ~91 kDa) and a putative truncated variant TRPV3 present in the treated sample. (B) Quantification of TRPV3 protein is presented as the normalized ratio over GAPDH expression.

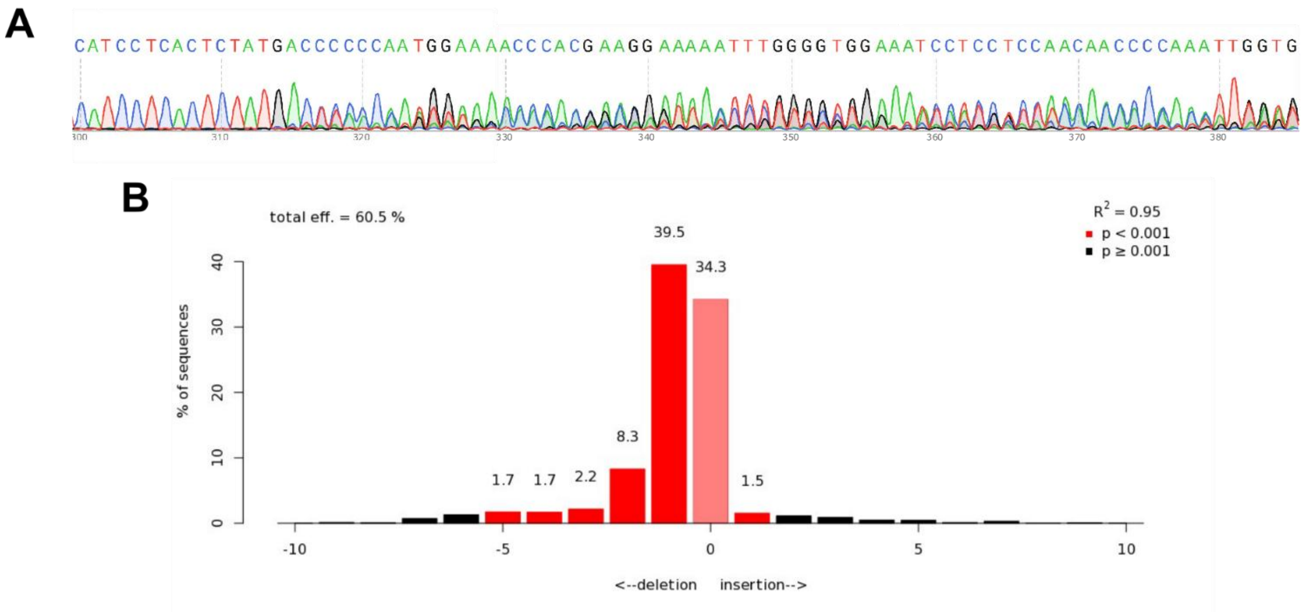

Figure S6. (A) Chromatogram of Trpv3 around the expected cut site of KERA-308 with SaCas9 and hygromycin resistance integrated via PiggyBack system. (B) TIDE analysis showing the position of the detected indels and the total efficiency of the SaCas9 disruption (60.5%).

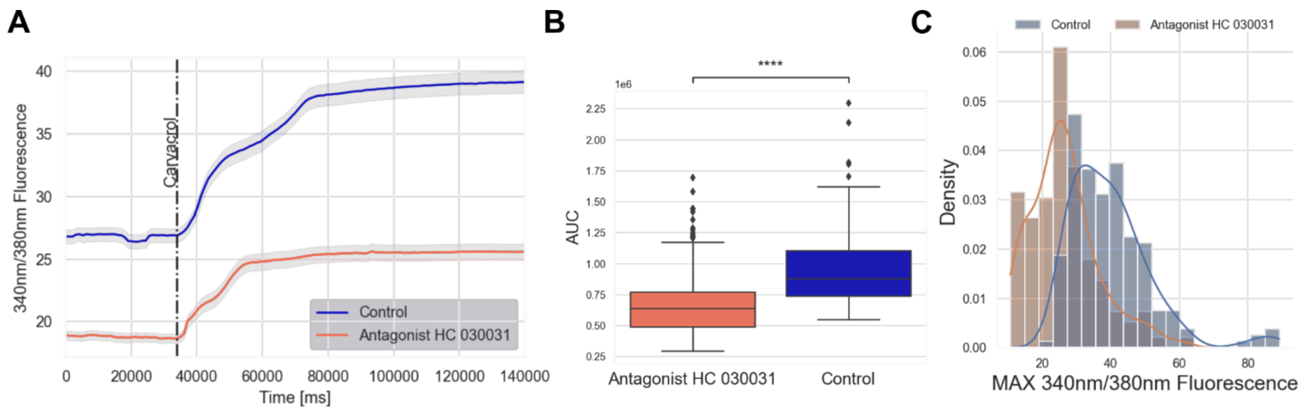

Figure S7. Antagonist HC 030031 effect on calcium imaging of Kera-308 (A) Average FURA2-AM 340nm/380nm fluorescence response ± SEM to carvacrol of KERA-308 cells in a control buffer (N=245) and in buffer supplemented with 50 μM of the TRPA1 antagonist HC 030031. (B) Mann-Whitney-Wilcoxon test with Bonferroni correction comparing the area under the curve for the first 30 seconds after carvacrol application between the two groups. P-value=1.034e-30. (C) Density plot of the maximal 340nm/380nm fluorescence response to carvacrol in control and HC 030031-treated groups.

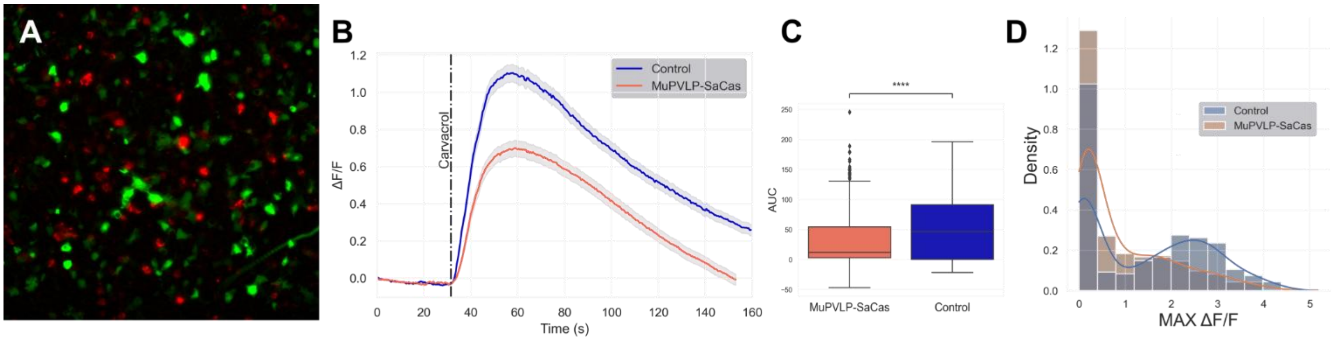

Figure S8. (A) Imaging of KERA-308 cells showing green fluorescence for GCaMP8s and red fluorescence for SaCas9-T2A-mCherry. (B) Average normalized fluorescence response (ΔF/F) ± SEM of control (N=574) and MuPVLP-SaCas9-treated (N=464) KERA-308 cells to carvacrol. (C) Mann-Whitney-Wilcoxon test with Bonferroni correction comparing the area under the curve for the first minute after carvacrol application. P_value=3.772e-05. (D) Density plot with kernel density estimation of the maximal normalized fluorescence (MAX ΔF/F) response to carvacrol for both groups.

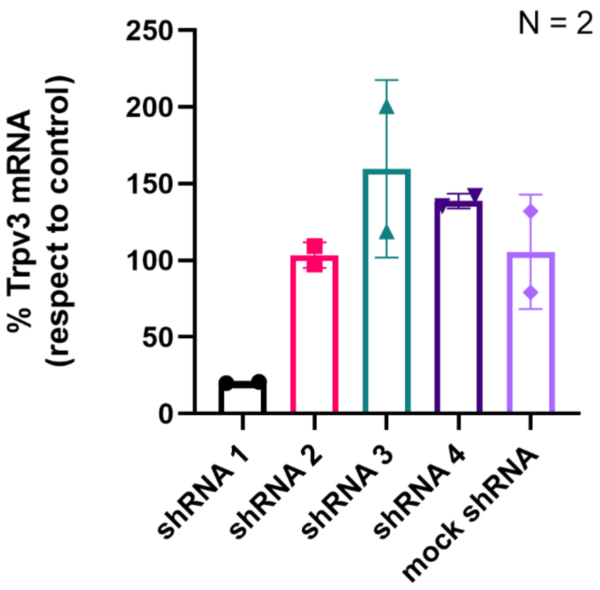

Figure S9. We evaluated four candidate shRNA against the Trpv3 mRNA in the KERA-308 cell line. shRNAs were cloned into a plasmid expressing YFP as a fluorescent reporter and delivered to KERA-308 via cell-free-assembled MuPVLP. Transduced KERA-308 were sorted via Fluorescence-Activated Cell Sorting (FACS) 5 days post-treatment based on YFP expression. The RNA was extracted from both the YFP+ and YFP- (control) populations and Trpv3 mRNA levels were quantified via relative qPCR with respect to GAPDH expression. The data is presented as the percentage of Trpv3 mRNA expression with respect to the YFP- population. We chose shRNA1 to perform all the experiments with MuPVLP.

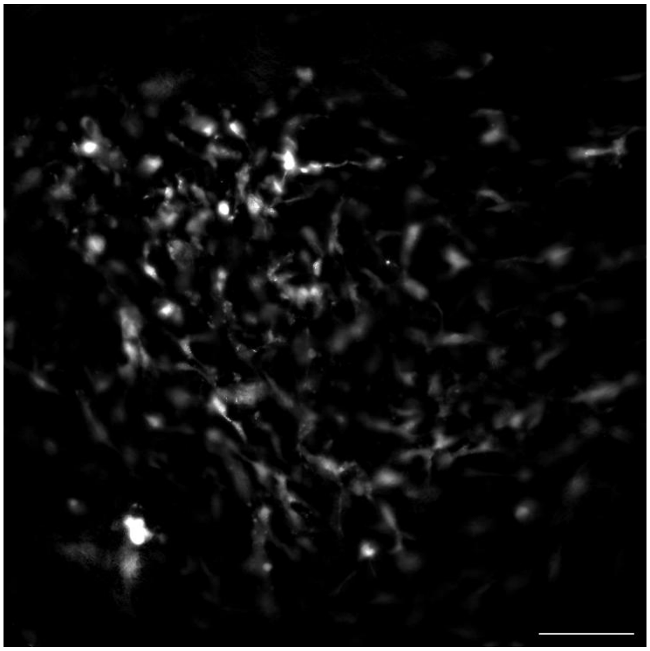

Figure S10. Representative image of an unsectioned skin equivalent sample transduced at 7 days in vitro with MuPVLP-TdTomato, captured on an inverted microscope with the red fluorescence filter 7 days post infection. Visual inspection suggested that only NIH 3T3 fibroblasts were transduced by MuPVLP-TdTomato. The white scale bar represents 50 µm.

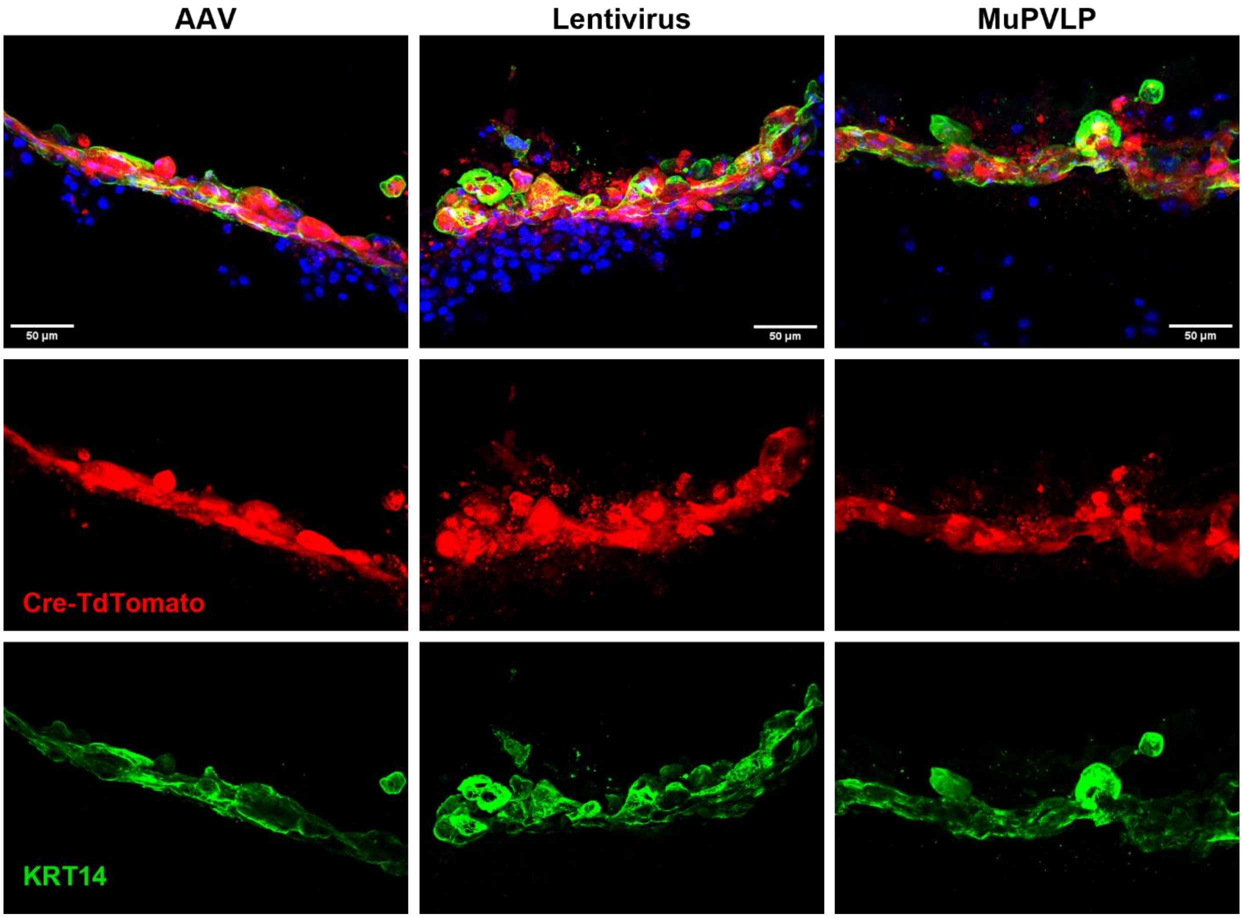

Figure S11. Confocal images of sections of skin equivalents transduced with either AAV1/2-Cre 10^6 MOI, Lentivirus-Cre 5 MOI, or MuPVLP-Cre 5000 MOI. Immunostaining for DAPI (blue), Keratin14 (green) and in red the endogenous expression of TdTomato consequent to Cre-recombination.

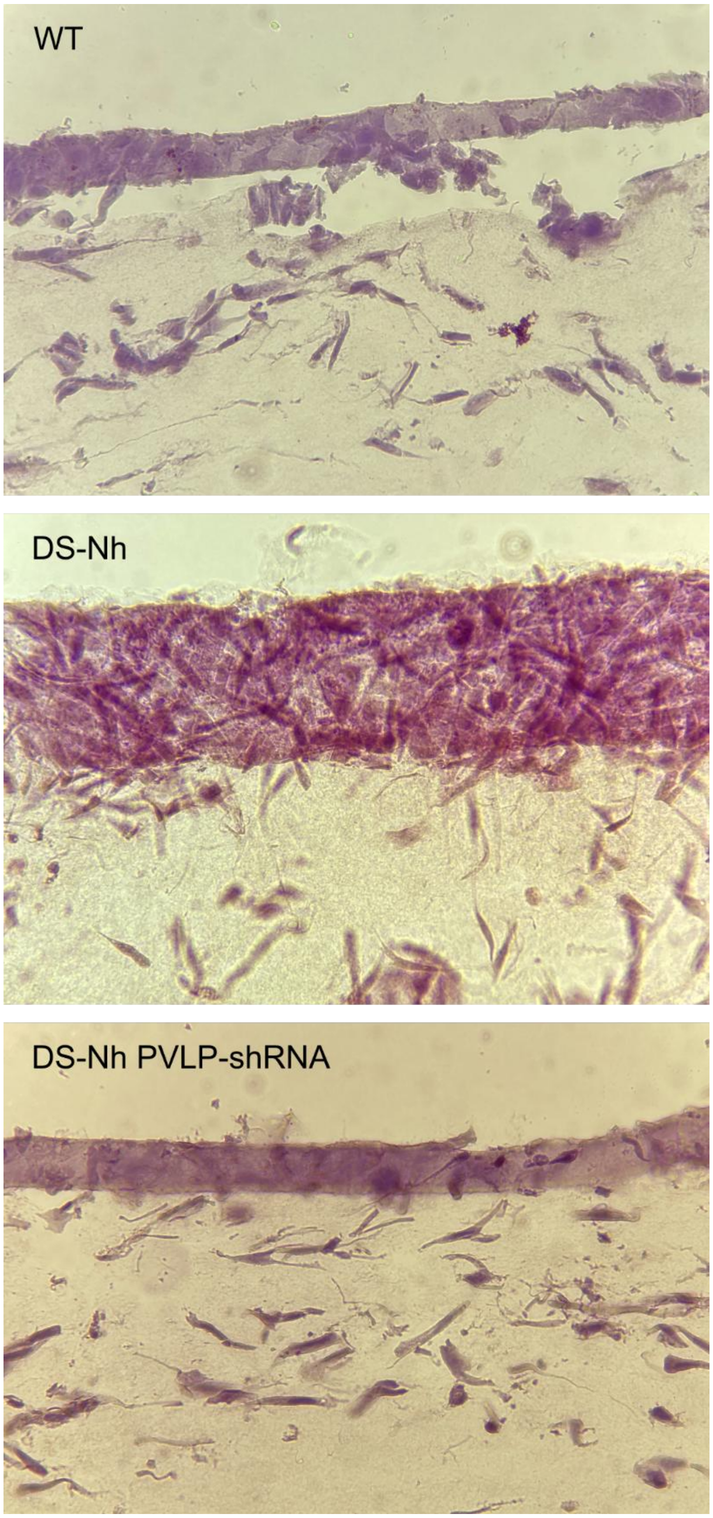

Figure S12. Hematoxylin and Eosin staining of WT, DS-Nh or MuPVLP-shRNA-treated DS-Nh skin equivalent acquired at inverted microscope 40x.

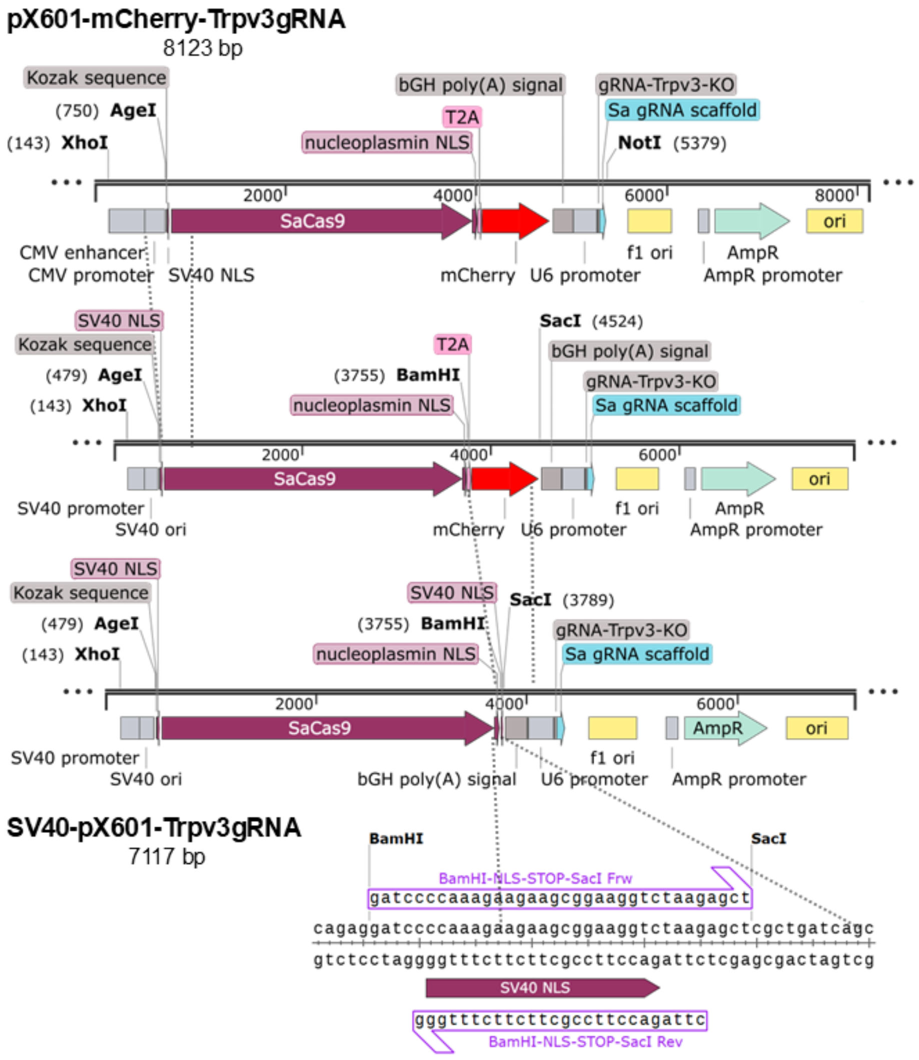

Figure S13. Cloning strategy for the pX601-mCherry-Trpv3gRNA plasmid. The first plasmid, pX601-mCherry-Trpv3gRNA, digested with XhoI and NotI enzymes, was used to prepare cell-free-assembled MuPVLP for experiments conducted on KERA-308 cells. The last plasmid, SV40-pX601-Trpv3gRNA, was utilized in experiments involving primary keratinocytes. To produce cell-assembled PVLP, we reduced the size of the plasmid by replacing the larger CMV promoter (584 bp) with the SV40 promoter (330 bp), also necessary for PVLP production. To further shrink the size of the plasmid from 7869 bp to 7117 bp, the mCherry reporter was removed, by digesting the plasmid with BamHI and SacI. The insert was designed to have the same BamHI and SacI restriction sites and to contain another SV40 Nuclear Localization Sequence (NLS) and a STOP codon.
